# Copper-Catalyzed Asymmetric Sulfonylative Desymmetrization of Glycerol

**DOI:** 10.3390/molecules27249025

**Published:** 2022-12-18

**Authors:** Kosuke Yamamoto, Keisuke Miyamoto, Mizuki Ueno, Yuki Takemoto, Masami Kuriyama, Osamu Onomura

**Affiliations:** Graduate School of Biomedical Sciences, Nagasaki University, 1-14 Bunkyo-machi, Nagasaki 852-8521, Japan

**Keywords:** copper catalysis, asymmetric reaction, desymmetrization, glycerol

## Abstract

Glycerol is the main side product in the biodiesel manufacturing process, and the development of glycerol valorization methods would indirectly contribute the sustainable biodiesel production and decarbonization. Transformation of glycerol to optically active C3 units would be one of the attractive routes for glycerol valorization. We herein present the asymmetric sulfonylative desymmetrization of glycerol by using a CuCN/(*R*,*R*)-PhBOX catalyst system to provide an optically active monosulfonylated glycerol in high efficiency. A high degree of enantioselectivity was achieved with a commercially available chiral ligand and an inexpensive carbonate base. The optically active monosulfonylated glycerol was successfully transformed into a C3 unit attached with differentially protected three hydroxy moieties. In addition, the synthetic utility of the present reaction was also demonstrated by the transformation of the monosulfonylated glycerol into an optically active synthetic ceramide, sphingolipid E.

## 1. Introduction

**Scheme 1:**
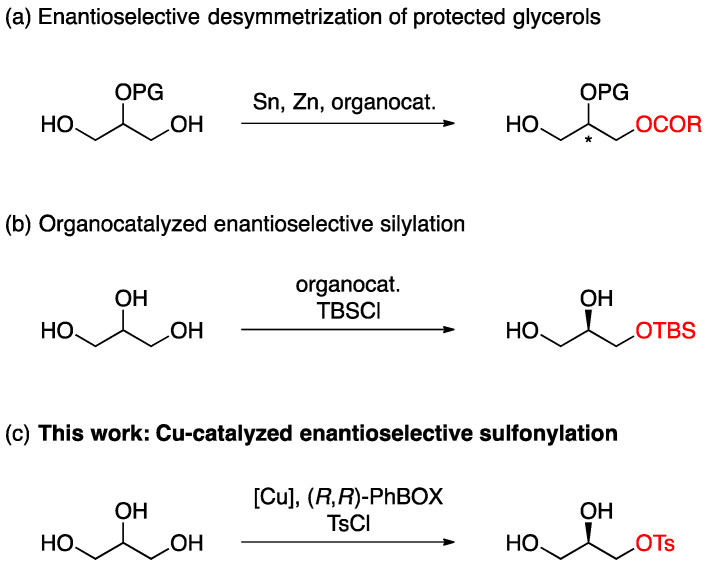
Asymmetric desymmetrization of glycerols. (**a**) Enantioselective desymmetrization of protected glycerols. (**b**) Organocatalyzed enantioselective silylation. (**c**) This work: Cu-catalyzed enantioselective sulfonylation. The asterisk denotes the chiral center.

The use of renewable energies instead of conventional fossil fuels has become a global trend from the perspective of expected fossil fuel depletion and global climate change. Biodiesel fuel, which is produced by the transesterification of vegetable or animal fats with methanol, has emerged as a promising alternative to petroleum-derived diesel fuel [1,2]. The biodiesel manufacturing process inevitably provides 10 wt% of glycerol (1,2,3-propanetriol) as the main side product, and an oversupply of the crude glycerol would be projected with the growing biodiesel market [3]. Thus, great effort has been devoted to the development of an efficient process for the transformation of glycerol to value-added commodity chemicals, which would indirectly contribute to sustainable biodiesel production [4,5,6,7]. Among them, optically active glycerol derivatives would be an attractive target in the field of glycerol valorization. Chiral glycerol derivatives such as glyceraldehyde and glycidyl tosylate are utilized as valuable C3 building blocks in medicinal [8,9,10,11,12] and synthetic organic chemistry [13,14,15,16,17,18]. A chiral pool approach is a traditional strategy to access enantiopure glycerol derivatives, but the need for multi-step transformations may be a major drawback [19,20,21,22,23]. Asymmetric desymmetrization of glycerol would be one of the most straightforward methods for chiral glycerol derivatives production. Several types of enzymes, i.e., lipase, kinase, and dehydrogenase/oxidase, have been successfully applied to this strategy, affording optically active glycerols with various enantioselectivities [24,25,26,27,28,29]. On the other hand, despite the recent development of the enantioselective desymmetrization [30,31] of 1,2-diols [32,33,34,35,36,37,38,39] and 1,3-diols [40,41,42,43,44,45,46,47,48], including C2-substituted glycerols [49,50,51,52,53], the non-enzymatic direct desymmetrization of glycerol is still a challenging task presumably due to an extremely high hydrophilic nature of glycerol. In this context, the use of 2-*O*-protected glycerol derivatives would be the most common strategy for the chemical desymmetrization of glycerol (Scheme 1a) [54,55,56,57]. In 2013, Tan et al. developed the first non-enzymatic direct desymmetrization of glycerol through organocatalyzed enantioselective silylation (Scheme 1b) [58]. In their protocol, the high enantioselectivity was achieved through the secondary kinetic resolution on the initially formed monosilylated glycerol. Very recently, the copper-catalyzed sulfonylative desymmetrization of glycerol using a non-commercially available ligand with silver carbonate was described in the Chinese patent [59]. Although the desired product was obtained with high enantioselectivity under copper-catalyzed conditions, the use of a commercially available chiral ligand and a non-precious metal base would be desirable from a practical and economical point of view [60,61,62]. Herein, we report the asymmetric desymmetrization of glycerol through sulfonylation with a Cu/(*R*,*R*)-PhBOX complex and sodium carbonate affording the optically active monosulfonylated glycerol in an excellent yield and enantioselectivity (Scheme 1c) [63].

## 2. Results and Discussion

For the initial attempt to optimize the enantioselective desymmetrization of glycerol, compound **1** was treated with *p*-toluenesulfonyl chloride (TsCl) in the presence of copper trifluoromethanesulfonate (Cu(OTf)_2_)/(*R*,*R*)-PhBOX and sodium carbonate in acetonitrile. Pleasingly, the desired monotosylated glycerol **2** was obtained in 91% yield with 83% ee (Table 1, entry 1). Using other carbonate salts, i.e., potassium carbonate and cesium carbonate, resulted in a decrease in both yield and enantioselectivity (entries 2 and 3). Organic bases were not suitable for the present transformation (entries 4 and 5). Next, other copper catalysts were examined to evaluate the catalytic activity in this reaction system. While CuCl provided **2** with a slightly lowered yield and enantioselectivity, CuBr and CuI exhibited a similar reactivity compared with Cu(OTf)_2_ (entries 6–8). The use of CuCN led to the formation of **2** in 83% yield with higher enantioselectivity, and the reaction concentration was able to be doubled without significant changes regarding both yield and enantioselectivity (entries 9–10). Pleasingly, we found that acetone was a better solvent choice to afford the desired product in an excellent yield and enantioselectivity (96% yield, 94% ee), and the concentration of 0.25 M was found to be suitable for the present reaction (entries 11–12). The catalyst loading was able to be reduced to 5 mol% without a significant decrease in the yield and enantioselectivity (entry 13). We also examined the feasibility of the gram-scale preparation of **2**. The reaction with 6.0 mmol of glycerol successfully provided the desired product **2** in 88% yield (1.30 g) with 93% ee (entry 14). Control experiments revealed that both the copper salt and the BOX ligand were essential to promote the tosylation of **1** (entries 15–16).

**Scheme 2:**
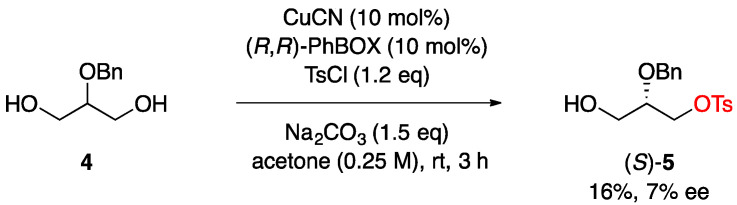
Enantioselective desymmetrization of 2-*O*-benzylglycerol.

In order to gain insight into the chemoselectivity of the present reaction, we performed competition studies with alcohol additives (Table 2). The addition of *n*-propanol (1.0 eq) led to a slight decrease in both yield and enantioselectivity, but the formation of *n*-propyl tosylate was not detected (entry 1 vs. entry 2). Moreover, selective sulfonylation of glycerol (**1**) over 1,2- and 1,3-diols was observed under the present reaction conditions, and the desired monosulfonylated glycerol **2** was obtained without a significant loss of enantioselectivity (entry 1 vs. entries 3–4). In addition, the reaction of 2-*O*-benzylglycerol (**4**) provided the corresponding monotosylated product **5** in a low yield with poor enantioselectivity (Scheme 2). These results indicated that the present reaction system would be highly selective for the glycerol transformation even in the presence of other alcohols, and the presence of a free 2-hydroxy moiety would play a crucial role in accelerating the tosylation with high asymmetric induction.

**Scheme 3:**
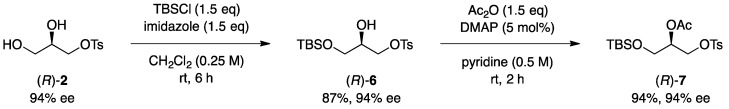
Synthesis of optically active glycerol derivatives.

With successful asymmetric desymmetrization of glycerol achieved, we then investigated the synthetic applications of the obtained optically active glycerol. First, the site-selective protection of the remained hydroxy groups in (*R*)-**2** was examined (Scheme 3). The primary hydroxy moiety was selectively protected by using *tert*-butyldimethylsilyl chloride (TBSCl) with imidazole, affording the corresponding product (*R*)-**6**. Acetylation of the secondary hydroxy group with Ac_2_O in the presence of a DMAP catalyst provided (*R*)-**7** in an excellent yield. Since each protective group would be removed by different deprotecting protocols, (*R*)-**7** would be potentially useful as a versatile chiral C3 building block.

**Scheme 4:**
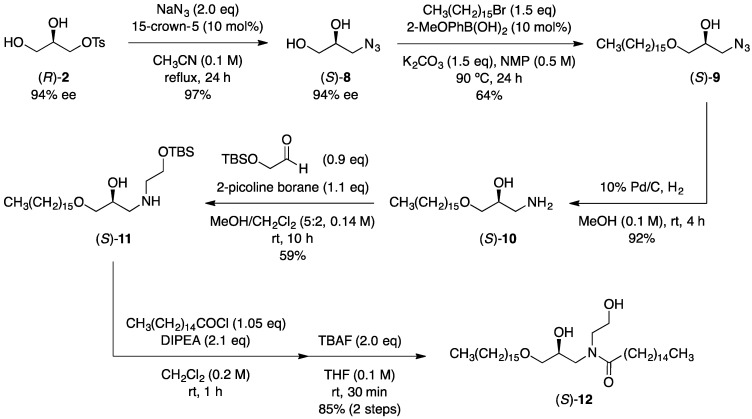
Synthesis of an optically active synthetic ceramide.

Next, we turned our attention to the application of the present transformation in the synthesis of an optically active synthetic ceramide. Ceramides are major components of the lamellar structure in stratum corneum lipids which protect the epidermis from excess transepidermal water loss and from the permeation of pathogens [64]. Interestingly, optically active natural ceramides showed different thermotropic behavior from racemic variants, and the lamellar liquid crystalline system containing optically active natural ceramides improved recovering effects of a water-holding ability and a barrier function of the skin [65]. Sphingolipid E (SLE) was a synthetic ceramide designed and synthesized by Kao Corporation as a structural analog of natural type 2 ceramide and was found to form a stable lamellar structure that exhibits a high water-holding ability [66]. Although SLE has been utilized as a racemic form, optically active SLE might affect the physicochemical property to form a lamellar structure and the interaction mode with water molecules. The synthesis of optically active SLE commenced with the introduction of nitrogen functionality to (*R*)-**2** (Scheme 4). The nucleophilic azide substitution of the tosyloxy group in (*R*)-**2** with sodium azide provided azide diol (*S*)-**8** in an excellent yield in the presence of 15-crown-5. The enantiomeric excess of (*S*)-**8** was determined after the tosylation of the primary hydroxy group, and no obvious racemization was observed in the azide substitution step. The boronic acid-catalyzed site-selective alkylation [67] of (*S*)-**8** with cetyl bromide followed by the Pd/C-catalyzed reduction of the azide group afforded the corresponding aminoalcohol (*S*)-**10**. *N*-Alkylated product (*S*)-**11** was successfully obtained by the reductive amination of (*S*)-**10** with TBS-protected glycolaldehyde using 2-picoline borane as a reductant. Finally, (*S*)-**11** was transformed into the optically active synthetic ceramide (*S*)-**12** via the amidation with palmitoyl chloride followed by the removal of the TBS group.

In conclusion, we have developed the copper-catalyzed asymmetric sulfonylative desymmetrization of glycerol. The reaction smoothly proceeded under mild reaction conditions with a commercially available (*R*,*R*)-PhBOX ligand and an inexpensive inorganic base, providing the optically active monotosylated glycerol derivative in a high yield with high enantiomeric excess. The synthetic utility of the present transformation was demonstrated by the preparation of an enantio-enriched C3 building block with three different types of protective groups. Moreover, the synthesis of the optically active synthetic ceramide was also achieved from the monotosylated glycerol in six steps without a notable loss of enantiopurity.

## 3. Materials and Methods

### 3.1. General

Unless otherwise noted, all reactions were performed under an argon atmosphere, and all reagents and solvents were used as received without further purification. Column chromatography was performed on *Fuji silysia* Chromatorex 60B silica gel. Melting points (mp) were measured with a *Yanako* Micro Melting Point Apparatus MP-J3 and reported without correction. Infrared (IR) spectra were recorded on a *Shimadzu* IRAffinity-1 spectrometer and expressed as frequency of absorption (cm^−1^). Optical rotations were measured with *JASCO* DIP-1000 or P-2200 spectrometers. ^1^H and ^13^C{^1^H} NMR spectra (Supplementary Materials) were recorded on *JEOL* JNM-AL400, JNM-ECZ400R (400 MHz for ^1^H NMR, 100 MHz for ^13^C{^1^H} NMR) or *Varian* NMR System 500PS SN (125 MHz for ^13^C{^1^H} NMR) spectrometers. Chemical shift values are expressed in parts per million (ppm) relative to internal TMS (*δ* 0.00 ppm for ^1^H NMR) or deuterated solvent peaks (*δ* 77.0 ppm (CDCl_3_) for ^13^C{^1^H} NMR). Abbreviations are as follows: s, singlet; d, doublet; t, triplet; q, quartet; m, multiplet; br, broad; app, apparent. Enantiomeric excess values were determined by chiral high-performance liquid chromatography (HPLC) analysis using *DAICEL* CHIRALPAK AD or AY-H columns. HPLC chromatograms (Supplementary Materials) were recorded on a CR8A CHROMATOPAC with an LC-20AT pump and SPD-20A UV detector (*Shimadzu*). High-resolution mass spectra (HRMS) were obtained on a *JEOL* JMS-700N (double-focusing magnetic sector mass analyzer) spectrometer with either the electron impact ionization (EI) or the fast atom bombardment (FAB) methods or a *JEOL* JMS-T100TD (TOF mass analyzer) spectrometer with either the direct analysis in real-time (DART) or the electrospray ionization (ESI) method.

### 3.2. Copper-Catalyzed Asymmetric Desymmetrization of Glycerol

A solution of (*R*,*R*)-PhBOX (33.4 mg, 0.10 mmol) and CuCN (8.96 mg, 0.10 mmol) in CH_2_Cl_2_ (4.0 mL) was stirred for 3 h at 40 °C. The reaction mixture was allowed to cool to room temperature and filtered into a round bottom flask using a cotton plug. After removal of the solvent under reduced pressure, the resulting solid was dried in vacuo for 30 min. To the round-bottom flask containing CuCN/(*R*,*R*)-PhBOX was added a solution of glycerol (92.1 mg, 1.0 mmol) in acetone (4.0 mL), and the resulting mixture was stirred for 10 min at room temperature. To the mixture was successively added Na_2_CO_3_ (159 mg, 1.5 mmol) and TsCl (229 mg, 1.2 mmol), and the mixture was stirred for 3 h at room temperature. The reaction was quenched with saturated aqueous NH_4_Cl, and the resulting mixture was extracted with AcOEt. Combined organic layers were washed with brine, dried over Na_2_SO_4_, filtered, and concentrated under reduced pressure. The crude product was purified by silica gel column chromatography (hexane/AcOEt = 1/2) to afford (*R*)-**2** (236 mg, 0.96 mmol, 96% yield).

*(R)-2,3-Dihydroxypropyl 4-methylbenzenesulfonate* ((*R*)-**2**). White solid; mp = 56–58 °C; [α]_D_^23^ −8.3 (*c* 1.00, MeOH) for 94% ee; ^1^H NMR (400 MHz, CDCl_3_): *δ* 7.81 (d, *J* = 8.5 Hz, 2H), 7.37 (d, *J* = 8.5 Hz, 2H), 4.13–4.06 (m, 2H), 3.99–3.93 (m, 1H), 3.74–3.69 (m, 1H), 3.66–3.60 (m, 1H), 2.51 (d, *J* = 5.5 Hz, 1H), 2.46 (s, 3H), 1.94 (t, *J* = 5.9 Hz, 1H); ^13^C{^1^H} NMR (100 MHz, CDCl_3_): *δ* 145.2, 132.2, 130.0, 127.9, 70.7, 69.6, 62.7, 21.6; IR (ATR): 3368, 2926, 1350, 1171, 968, 812 cm^−1^; HRMS (EI) *m*/*z*: [M]^+^ calcd for C_10_H_14_O_5_S 246.0562, found 254.0562; HPLC analysis: Chiralpak AY-H, hexane/EtOH = 2/1, flow rate 1.0 mL/min, wavelength 254 nm, *t*_R_ 13.5 min (minor) and 15.6 min (major). The absolute configuration of **2** was established by comparing the sign of the specific rotation of **2** with the literature value ([α]_D_^22^ −9.3 (*c* 4.99, MeOH) for (*R*)-**2**) [68].

### 3.3. Large-Scale Experiment

A solution of (*R*,*R*)-PhBOX (200 mg, 0.60 mmol) and CuCN (53.7 mg, 0.60 mmol) in CH_2_Cl_2_ (24 mL) was stirred for 3 h at 40 °C. The reaction mixture was allowed to cool to room temperature and filtered into a round bottom flask using a cotton plug. After removal of the solvent under reduced pressure, the resulting solid was dried in vacuo for 1 h. To the round-bottom flask containing CuCN/(*R*,*R*)-PhBOX was added a solution of glycerol (553 mg, 6.0 mmol) in acetone (24 mL), and the resulting mixture was stirred for 10 min at room temperature. To the mixture was successively added Na_2_CO_3_ (950 mg, 9.0 mmol) and TsCl (1.37 g, 7.2 mmol), and the mixture was stirred for 9 h at room temperature. The reaction was quenched with saturated aqueous NH_4_Cl, and the resulting mixture was extracted with AcOEt. Combined organic layers were washed with brine, dried over Na_2_SO_4_, filtered, and concentrated under reduced pressure. The crude product was purified by silica gel column chromatography (hexane/AcOEt = 1/2) to afford (*R*)-**2** (1.30 g, 5.28 mmol, 88% yield, 93% ee).

### 3.4. Copper-Catalyzed Asymmetric Desymmetrization of 2-O-Benzylglycerol

The reaction was performed using 2-*O*-benzylglycerol (**4**, 182 mg, 1.0 mmol) [69] according to the procedure described in Section 3.2. Silica gel column chromatography (hexane/acetone = 3/1) to afford (*S*)-**5** (52.6 mg, 0.16 mmol, 16% yield).

*(S)-2-(Benzyloxy)-3-hydroxypropyl 4-methylbenzenesulfonate* ((*S*)-**5**). colorless oil; [α]_D_^21^ −2.1 (*c* 1.00, CHCl_3_) for 7% ee; ^1^H NMR (400 MHz, CDCl_3_): *δ* 7.78 (d, *J* = 8.2 Hz, 2H), 7.36–7.27 (m, 7H), 4.63 (d, *J* = 11.7 Hz, 1H), 4.55 (d, *J* = 11.7 Hz, 1H), 4.17–4.10 (m, 2H), 3.75–3.67 (m, 2H), 3.62–3.56 (m, 1H), 2.45 (s, 3H), 1.80 (t, *J* = 6.3 Hz, 1H); ^13^C{^1^H} NMR (125 MHz, CDCl_3_): *δ* 145.0, 137.5, 132.6, 129.9, 128.5, 128.1, 128.0, 127.9, 76.7, 72.5, 68.7, 61.4, 21.7; IR (ATR): 3445, 3032, 2879, 1597, 1354, 1173, 974 cm^−1^; HRMS (ESI) *m*/*z*: [M + Na]^+^ calcd for C_17_H_20_NaO_5_S 359.0929, found 359.0922; HPLC analysis: Chiralpak AD, hexane/EtOH = 3/1, flow rate 1.0 mL/min, wavelength 254 nm, *t*_R_ 7.1 min (minor) and 8.8 min (major). The absolute configuration of **5** was established by comparing the sign of the specific rotation of **5** with the literature value ([α]_D_^22^ +29.5 (*c* 1.01, CHCl_3_) for (*R*)-**5**) [70].

### 3.5. Synthesis of Optically Active Glycerol Derivatives

*(R)-3-((tert-Butyldimethylsilyl)oxy)-2-hydroxypropyl 4-methylbenzenesulfonate* ((*R*)-**6**). To a solution of (*R*)-**2** (134 mg, 0.54 mmol) in CH_2_Cl_2_ (2.2 mL) was successively added imidazole (55.8 mg, 0.82 mmol) and TBSCl (123 mg, 0.82 mmol) at room temperature. After stirring for 6 h at the same temperature, the reaction was quenched with H_2_O. The resulting mixture was extracted with AcOEt. The combined organic layers were dried over Na_2_SO_4_, filtered, and concentrated under reduced pressure. The residue was purified by silica gel column chromatography (hexane/AcOEt = 2/1) to afford (*R*)-**6** (103 mg, 0.287 mmol, 87% yield) as a colorless oil; [α]_D_^27^ −12.4 (*c* 1.01, AcOEt) for 94% ee; ^1^H NMR (400 MHz, CDCl_3_): *δ* 7.82–7.79 (m, 2H), 7.37–7.34 (m, 2H), 4.07 (dd, *J* = 9.9, 5.6 Hz, 1H), 4.01 (dd, *J* = 9.9, 5.6 Hz, 1H), 3.87–3.83 (m, 1H), 3.66–3.59 (m, 2H), 2.45 (s, 3H), 2.40 (d, *J* = 6.2 Hz, 1H), 0.85 (s, 9H), 0.039 (s, 3H), 0.035 (s, 3H); ^13^C{^1^H} NMR (100 MHz, CDCl_3_): *δ* 145.0, 132.6, 129.9, 128.0, 70.0, 69.2, 62.8, 25.7, 21.6, 18.1, −5.6; IR (ATR): 3537, 2930, 2857, 1360, 1252, 1175, 980, 833 cm^−1^; HRMS (EI) *m*/*z*: [M]^+^ calcd for C_16_H_28_O_5_SSi 360.1427, found 360.1429. HPLC analysis: Chiralpak AY-H, hexane/*i*-PrOH = 10/1, flow rate 1.0 mL/min, wavelength 254 nm, *t*_R_ 22.4 min (minor) and 25.3 min (major).

*(R)-1-((tert-Butyldimethylsilyl)oxy)-3-(tosyloxy)propan-2-yl acetate* ((*R*)-**7**). To a solution of (*R*)-**6** (144 mg, 0.40 mmol) in pyridine (0.80 mL) was successively added DMAP (2.44 mg, 0.020 mmol) and Ac_2_O (49.0 mg, 0.48 mmol) at room temperature. After stirring for 2 h at the same temperature, the reaction mixture was diluted with toluene (5.0 mL) and concentrated under reduced pressure. The residue was purified by silica gel column chromatography (hexane/AcOEt = 5/1) to afford (*R*)-**7** (151 mg, 0.378 mmol, 94% yield) as a colorless oil. [α]_D_^27^ −2.2 (*c* 1.24, AcOEt) for 94% ee; ^1^H NMR (400 MHz, CDCl_3_): *δ* 7.79 (d, *J* = 8.2 Hz, 2H), 7.35 (d, *J* = 8.0 Hz, 2H), 4.96–4.91 (m, 1H), 4.22 (dd, *J* = 10.7, 3.7 Hz, 1H), 4.17 (dd, *J* = 10.9, 5.4 Hz, 1H), 3.70–3.63 (m, 2H), 2.45 (s, 3H), 2.00 (s, 3H), 0.83 (s, 9H), 0.01 (s, 6H); ^13^C{^1^H} NMR (100 MHz, CDCl_3_): *δ* 170.0, 144.9, 132.7, 129.8, 128.0, 71.3, 67.8, 60.5, 25.6, 21.6, 20.8, 18.1, −5.58, −5.62; IR (ATR): 1744, 1362, 1233, 1175, 988, 833 cm^−1^; HRMS (EI) *m*/*z*: [M]^+^ calcd for C_18_H_30_O_6_SSi 402.1532, found 402.1533; HPLC analysis: Chiralpak AD, hexane/EtOH = 20/1, flow rate 1.0 mL/min, wavelength 254 nm, *t*_R_ 4.8 min (major) and 6.5 min (minor).

### 3.6. Synthesis of an Optically Active Synthetic Ceramide

*(S)-3-Azidopropane-1,2-diol* ((*S*)-**8**). To a solution of (*R*)-**2** (246 mg, 1.0 mmol) in CH_3_CN (10 mL) was successively added 15-crown-5 (22.1 mg, 0.10 mmol) and NaN_3_ (130 mg, 2.0 mmol) at room temperature. After refluxing for 24 h, the reaction mixture was filtered using celite, and the filtrate was concentrated under reduced pressure. The residue was purified by silica gel column chromatography (CH_2_Cl_2_/MeOH = 12/1) to afford (*S*)-**8** (114 mg, 0.974 mmol, 97% yield) as a colorless oil; [α]_D_^23^ −16.2 (*c* 1.10, MeOH); ^1^H NMR (400 MHz, CDCl_3_): *δ* 3.92–3.86 (m, 1H), 3.74–3.72 (m, 1H), 3.65–3.61 (m, 1H), 3.47–3.38 (m, 2H), 2.49 (d, *J* = 4.1 Hz, 1H), 1.95 (br s, 1H); ^13^C{^1^H} NMR (100 MHz, CDCl_3_): *δ* 70.9, 63.9, 53.4; IR (ATR): 3333, 2924, 2855, 2093, 1443, 1272, 1103, 1038, 928 cm^−1^; HRMS (EI) *m*/*z*: [M]^+^ calcd for C_3_H_7_N_3_O_2_ 117.0538, found 117.0546. The absolute configuration of **8** was established by comparing the sign of the specific rotation of **8** with the literature value ([α]_D_^20^ −17.4 (*c* 1.00, MeOH) for (*S*)-**8**) [71].

*(S)-1-Azido-3-(hexadecyloxy)propan-2-ol* ((*S*)-**9**). To a solution of (*S*)-**8** (46.8 mg, 0.40 mmol) in *N*-methylpyrrolidone (0.80 mL) was successively added 2-methoxyphenylboronic acid (6.0 mg, 0.040 mmol) and K_2_CO_3_ (82.9 mg, 0.60 mmol). The resulting mixture was stirred for 30 min at room temperature, and then cetyl bromide (183 mg, 0.60 mmol) was added. After stirring for 24 h at 95 °C, the reaction mixture was diluted with H_2_O and extracted with AcOEt. The combined organic layers were washed with brine, dried over Na_2_SO_4_, filtered, and concentrated under reduced pressure. The residue was purified by silica gel column chromatography (hexane/Et_2_O = 8/2) to afford (*S*)-**9** (87.3 mg, 0.256 mmol, 64% yield) as a white solid; mp = 37–39 °C; [α]_D_^24^ −11.8 (*c* 1.00, MeOH); ^1^H NMR (400 MHz, CDCl_3_): *δ* 3.97–3.91 (m, 1H), 3.51–3.33 (m, 6H), 2.42 (d, *J* = 5.0 Hz, 1H), 1.61–1.54 (m, 2H), 1.32–1.26 (m, 26H), 0.88 (t, *J* = 6.9 Hz, 3H); ^13^C{^1^H} NMR (100 MHz, CDCl_3_): *δ* 71.8, 71.7, 69.6, 53.5, 31.9, 29.68, 29.66, 29.64, 29.59, 29.57, 29.5, 29.4, 29.3, 26.1, 22.7, 14.1; IR (ATR): 3429, 2914, 2876, 2846, 2088, 1466, 1337, 1290, 1113, 989 cm^−1^; HRMS (DART) *m*/*z*: [M + H]^+^ calcd for C_19_H_40_N_3_O_2_ 342.3121, found 342.3171.

*(S)-1-Amino-3-(hexadecyloxy)propan-2-ol* ((*S*)-**10**). To a reaction vessel charged with 10% Pd/C (24.9 mg, 10% w/w) was added a solution of (*S*)-**9** (249 mg, 0.73 mmol) in MeOH (7.3 mL) at room temperature under argon atmosphere. The reaction vessel was charged with H_2_ gas, and then the mixture was stirred for 4 h at room temperature. The reaction mixture was filtered using celite, and then the filtrate was concentrated under reduced pressure. The residue was dissolved in 10% aqueous HCl and washed with AcOEt. The aqueous layer was basified with saturated aqueous NaHCO_3_ and then extracted with CHCl_3_. The combined organic layers were dried over Na_2_SO_4_, filtered, and concentrated under reduced pressure to afford (*S*)-**10** (211 mg, 0.67 mmol, 92% yield) as a white solid; mp = 60–61 °C; [α]_D_^24^ −3.2 (*c* 0.50, CHCl_3_); ^1^H NMR (400 MHz, CDCl_3_): *δ* 3.76–3.70 (m, 1H), 3.50–3.43 (m, 3H), 3.38 (dd, *J* = 9.5, 6.5 Hz, 1H), 2.83 (dd, *J* = 12.7, 3.1 Hz, 1H), 2.72 (dd, *J* = 12.5, 6.7 Hz, 1H), 1.61–1.54 (m, 2H), 1.32–1.26 (m, 26H), 0.88 (t, *J* = 6.7 Hz, 3H); ^13^C{^1^H} NMR (100 MHz, CDCl_3_): *δ* 73.0, 71.7, 71.1, 44.4, 31.9, 29.7, 29.64, 29.59, 29.58, 29.5, 29.3, 26.1, 22.7, 14.1; IR (ATR): 2912, 2827, 1470, 1130, 1032, 924 cm^−1^; HRMS (FAB) *m*/*z*: [M + H]^+^ calcd for C_19_H_42_NO_2_ 316.3216, found 316.3200.

*(S)-2,2,3,3-Tetramethyl-4,11-dioxa-7-aza-3-silaheptacosan-9-ol* ((*S*)-**11**). To a solution of 2-(*tert*-butyldimethylsilyloxy)acetaldehyde (34.9 mg, 0.20 mmol) [72] in MeOH/CH_2_Cl_2_ (5:2, 1.4 mL) was added (*S*)-**10** (69.4 mg, 0.22 mmol) at room temperature. After stirring for 10 min at the same temperature, 2-picoline borane (256 mg, 0.24 mmol) was added, and then the reaction mixture was stirred for an additional 10 h. The reaction mixture was diluted with H_2_O and extracted with CH_2_Cl_2_. The combined organic layers were dried over Na_2_SO_4_, filtered, and concentrated under reduced pressure. The residue was purified by silica gel column chromatography (CH_2_Cl_2_/MeOH = 12/1) to afford (*S*)-**11** (55.8 mg, 0.117 mmol, 59% yield) as a colorless amorphous; [α]_D_^25^ −3.6 (*c* 1.00, CHCl_3_); ^1^H NMR (400 MHz, CDCl_3_): *δ* 3.86–3.81 (m, 1H), 3.75–3.67 (m, 2H), 3.49–3.39 (m, 4H), 2.78–2.64 (m, 4H), 1.60–1.54 (m, 2H), 1.33–1.25 (m, 26H), 0.90–0.86 (m, 12H), 0.06 (s, 6H); ^13^C{^1^H} NMR (100 MHz, CDCl_3_): *δ* 73.3, 71.7, 68.7, 62.2, 51.7, 51.5, 31.9, 29.7, 29.62, 29.58, 29.5, 29.3, 26.1, 25.9, 22.7, 18.3, 14.1, −5.4; IR (ATR): 2914, 2849, 1472, 1464, 1256, 1119, 1080, 968, 937, 831 cm^−1^; HRMS (FAB) *m*/*z*: [M + H]^+^ calcd for C_27_H_60_NO_3_Si 473.4342, found 473.4300.

*(S)-N-(3-(Hexadecyloxy)-2-hydroxypropyl)-N-(2-hydroxyethyl)palmitamide* ((*S*)-**12**). To a solution of (*S*)-**11** (135 mg, 0.28 mmol) and *i*-Pr_2_NEt (77.0 mg, 0.60 mmol) in CH_2_Cl_2_ (1.4 mL) was added palmitoyl chloride (81.9 mg, 0.30 mmol) at room temperature. After stirring for 1 h at the same temperature, all volatile was removed under reduced pressure. The residue was dissolved in THF (2.8 mL), and then a 1.0 M solution of TBAF in THF (0.57 mL) was added at room temperature. After stirring for 30 min at the same temperature, the reaction mixture was diluted with H_2_O and extracted with CHCl_3_. The combined organic layers were dried over Na_2_SO_4_, filtered, and concentrated under reduced pressure. The residue was purified by silica gel column chromatography (hexane/AcOEt = 1/1) to afford (*S*)-**12** (144 mg, 0.241 mmol, 85% yield) as a white solid; mp = 67–68 °C; [α]_D_^25^ −4.4 (*c* 1.00, CHCl_3_); ^1^H NMR (400 MHz, CDCl_3_, mixture of rotamers): *δ* 4.16–3.93 (m, 1H), 3.85–3.74 (m, 2H), 3.67–3.25 (m, 8H), 2.46–2.30 (m, 2H), 1.67–1.52 (m, 4H), 1.28 (m, 50H), 0.88 (app t, *J* = 6.9 Hz, 6H). ^13^C{^1^H} NMR (100 MHz, CDCl_3_): *δ* 175.8, 72.4, 72.1, 71.8, 71.6, 69.8, 69.4, 61.7, 60.6, 53.3, 52.5, 51.4, 51.1, 33.6, 33.5, 31.9, 29.7, 29.62, 29.60, 29.59, 29.56, 29.53, 29.47, 29.44, 29.42, 29.3, 26.1, 26.0, 25.29, 25.26, 22.7, 14.1; IR (ATR): 3320, 2916, 2849, 1611, 1464, 1437, 1375, 1306, 1290, 1261, 1206, 1165, 1109, 1094, 1059, 1040, 955, 845, 814 cm^−1^; HRMS (FAB) *m*/*z*: [M + H]^+^ calcd for C_37_H_76_NO_4_ 598.5773, found 598.5800.

### 3.7. Tosylation of Azide Diol (S)-***8***

*(S)-3-Azido-2-hydroxypropyl 4-methylbenzenesulfonate* ((*S*)-**8′**). To a solution of (*S*)-**8** (23.4 mg, 0.20 mmol) in CH_3_CN (0.80 mL) was added pyridine (23.7 mg, 0.30 mmol) and TsCl (57.2 mg, 0.30 mmol) at room temperature. After stirring for 10 h at the same temperature, the reaction was quenched with H_2_O, and the resulting mixture was extracted with AcOEt. The combined organic layers were washed with brine, dried over Na_2_SO_4_, filtered, and concentrated under reduced pressure. The residue was purified by silica gel column chromatography (hexane/AcOEt = 2/1) to afford (*S*)-**8′** (30.3 mg, 0.112 mmol, 56% yield) as a colorless oil; [α]_D_^27^ −17.5 (*c* 1.00, AcOEt) for 94% ee; ^1^H NMR (400 MHz, CDCl_3_): *δ* 7.81 (d, *J* = 8.3 Hz, 2H), 7.38 (d, *J* = 8.3 Hz, 2H), 4.09–3.98 (m, 3H), 3.43 (dd, *J* = 12.9, 4.6 Hz, 1H), 3.38 (dd, *J* = 12.7, 5.4 Hz, 1H), 2.47 (s, 3H), 2.40 (d, *J* = 5.4 Hz, 1H); ^13^C{^1^H} NMR (100 MHz, CDCl_3_): *δ* 145.4, 132.3, 130.0, 128.0, 70.5, 68.5, 52.7; IR (ATR): 3462, 2100, 1597, 1352, 1173, 1096, 982 cm^−1^; HRMS (EI) *m*/*z*: [M]^+^ calcd for C_10_H_13_N_3_O_4_S 271.0627, found 271.0622; HPLC analysis: Chiralpak AY-H, hexane/EtOH = 6/1, flow rate 1.0 mL/min, wavelength 254 nm, *t*_R_ 28.9 min (minor) and 31.3 min (major).

## Figures and Tables

**Table 1:** Optimization of reaction conditions ^1^.

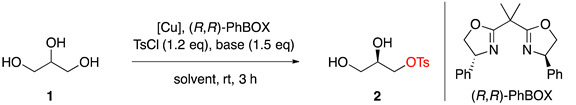					
**Entry**	**[Cu]**	**Base**	**Solvent**	**Yield (%) ^2^**	**ee (%) ^3^**
1	Cu(OTf)_2_	Na_2_CO_3_	CH_3_CN	91	83
2	Cu(OTf)_2_	K_2_CO_3_	CH_3_CN	62	65
3 ^4^	Cu(OTf)_2_	Cs_2_CO_3_	CH_3_CN	16	47
4 ^4^	Cu(OTf)_2_	pyridine	CH_3_CN	41	*rac*
5 ^4^	Cu(OTf)_2_	DIPEA	CH_3_CN	51	43
6	CuCl	Na_2_CO_3_	CH_3_CN	73	79
7	CuBr	Na_2_CO_3_	CH_3_CN	89	84
8	CuI	Na_2_CO_3_	CH_3_CN	80	87
9	CuCN	Na_2_CO_3_	CH_3_CN	83	90
10 ^5^	CuCN	Na_2_CO_3_	CH_3_CN	82	90
11 ^5^	CuCN	Na_2_CO_3_	acetone	96	94
12 ^6^	CuCN	Na_2_CO_3_	acetone	93	89
13 ^5,7^	CuCN	Na_2_CO_3_	acetone	83	91
14 ^5,8^	CuCN	Na_2_CO_3_	acetone	88	93
15 ^5^	–	Na_2_CO_3_	acetone	trace	–
16 ^5,9^	CuCN	Na_2_CO_3_	acetone	3	*rac*

^1^ Reaction conditions: **1** (1.0 mmol), TsCl (1.2 mmol), [Cu] (0.1 mmol), (*R*,*R*)-PhBOX (0.1 mmol), base (1.5 mmol), solvent (0.125 M), rt, 3 h. ^2^ Isolated yield after column chromatography. ^3^ Determined by chiral HPLC analysis. ^4^ Reaction time (12 h). ^5^ Solvent (0.25 M). ^6^ Solvent (0.5 M). ^7^ CuCN (0.05 mmol), (*R*,*R*)-PhBOX (0.05 mmol), 10 h. ^8^ **1** (6.0 mmol), 9 h. ^9^ The reaction was carried out in the absence of (*R*,*R*)-PhBOX.

**Table 2:** Competition studies ^1^.

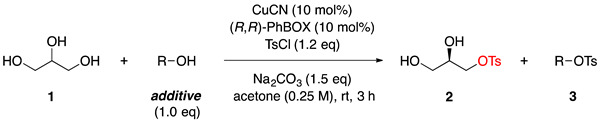				
**Entry**	**Additive**	**Yield of 2 (%) ^2^**	**ee of 2 (%) ^3^**	**Yield of 3 (%) ^2^**
1	none	96	94	n.d.
2	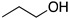	81	85	n.d.
3	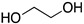	93	94	n.d.
4	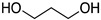	91	95	n.d.

^1^ Reaction conditions: **1** (1.0 mmol), additive (1.0 mmol), TsCl (1.2 mmol), CuCN (0.1 mmol), (*R*,*R*)-PhBOX (0.1 mmol), Na_2_CO_3_ (1.5 mmol), acetone (0.25 M), rt, 3 h. ^2^ Isolated yield after column chromatography. n.d. = not detected. ^3^ Determined by chiral HPLC analysis.

## Data Availability

The data presented in this study are available in Supplementary Material.

## Supplementary Materials

The following supporting information can be downloaded at: https://www.mdpi.com/article/10.3390/molecules27249025/s1, Figures S1–S10: Copies of ^1^H and ^13^C{^1^H} NMR spectra of compounds **2–12**; Figures S11–S15: Chiral HPLC chromatogram of compounds **2**, **5**, **6**, **7**, and **8′**.

## Sample Availability

Samples of the compounds are not available from the authors.
