# Supplementary material for: Copper-Catalyzed Asymmetric Sulfonylative Desymmetrization of Glycerol
Source: Molecules. 2022 Dec 18;27(24):9025. doi: 10.3390/molecules27249025 (PMC9780796; doi:10.3390/molecules27249025)
Supplement: Supplementary file 1 [file molecules-27-09025-s001.zip › molecules-2090391-supplementary.pdf]

Copper-Catalyzed Asymmetric Sulfonylative Desymmetrization of Glycerol

Kosuke Yamamoto, Keisuke Miyamoto, Mizuki Ueno, Yuki Takemoto, Masami Kuriyama, and Osamu Onomura*

Graduate School of Biomedical Sciences, Nagasaki University, 1-14 Bunkyo-machi, Nagasaki 852-8521, Japan

*onomura@nagasaki-u.ac.jp

Supporting Information

Table of Contents

1. ^{1}H and $^{13}C\{^{1}H\}$ NMR spectra S2-S11

2. Chiral HPLC chromatograms S12-S14

1. ^{1}H and $^{13}C\{^{1}H\}$ NMR spectra

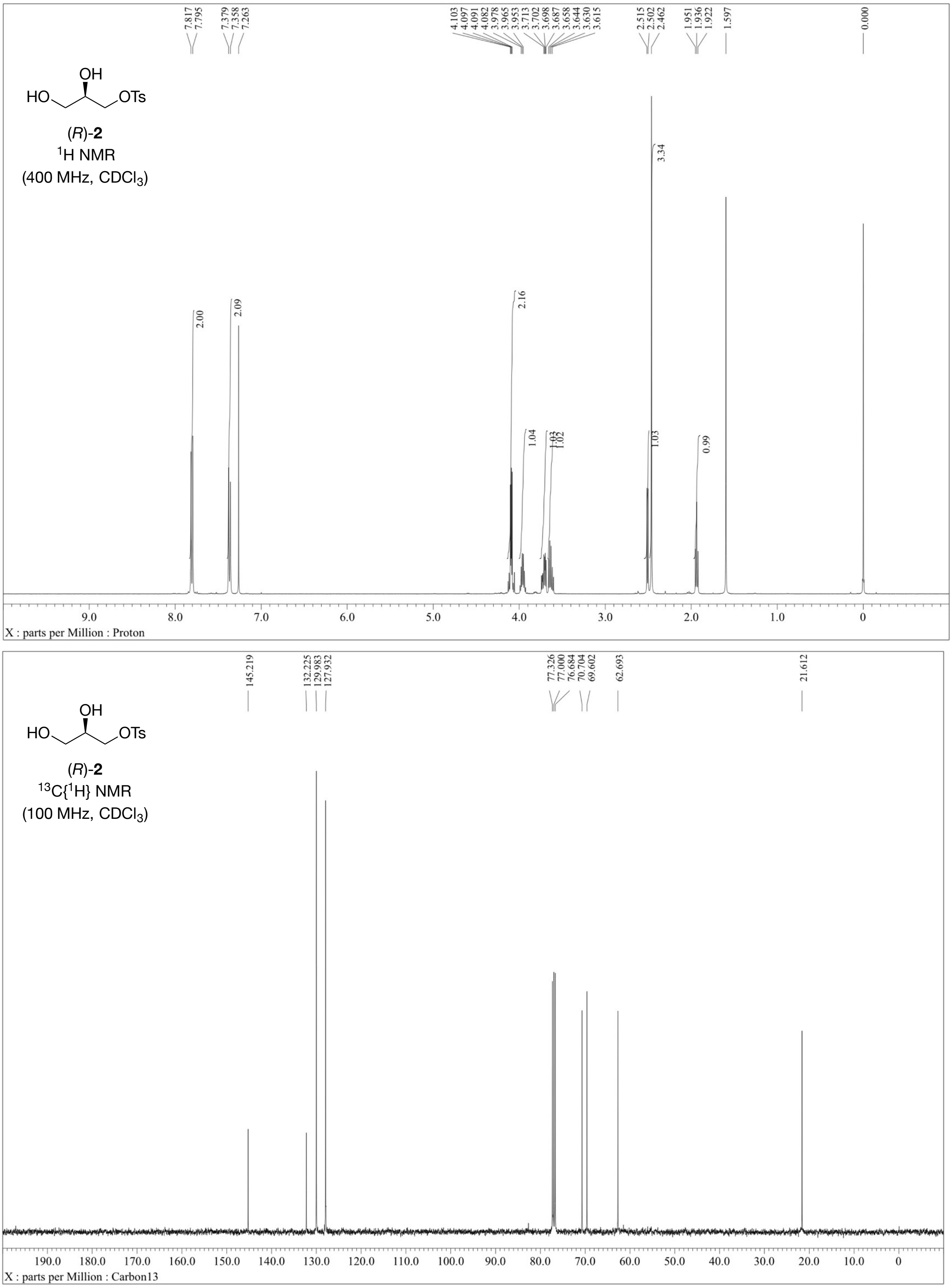

Figure S1. ^{1}H and $^{13}C\{^{1}H\}$ NMR spectra of (*R*)-**2**.

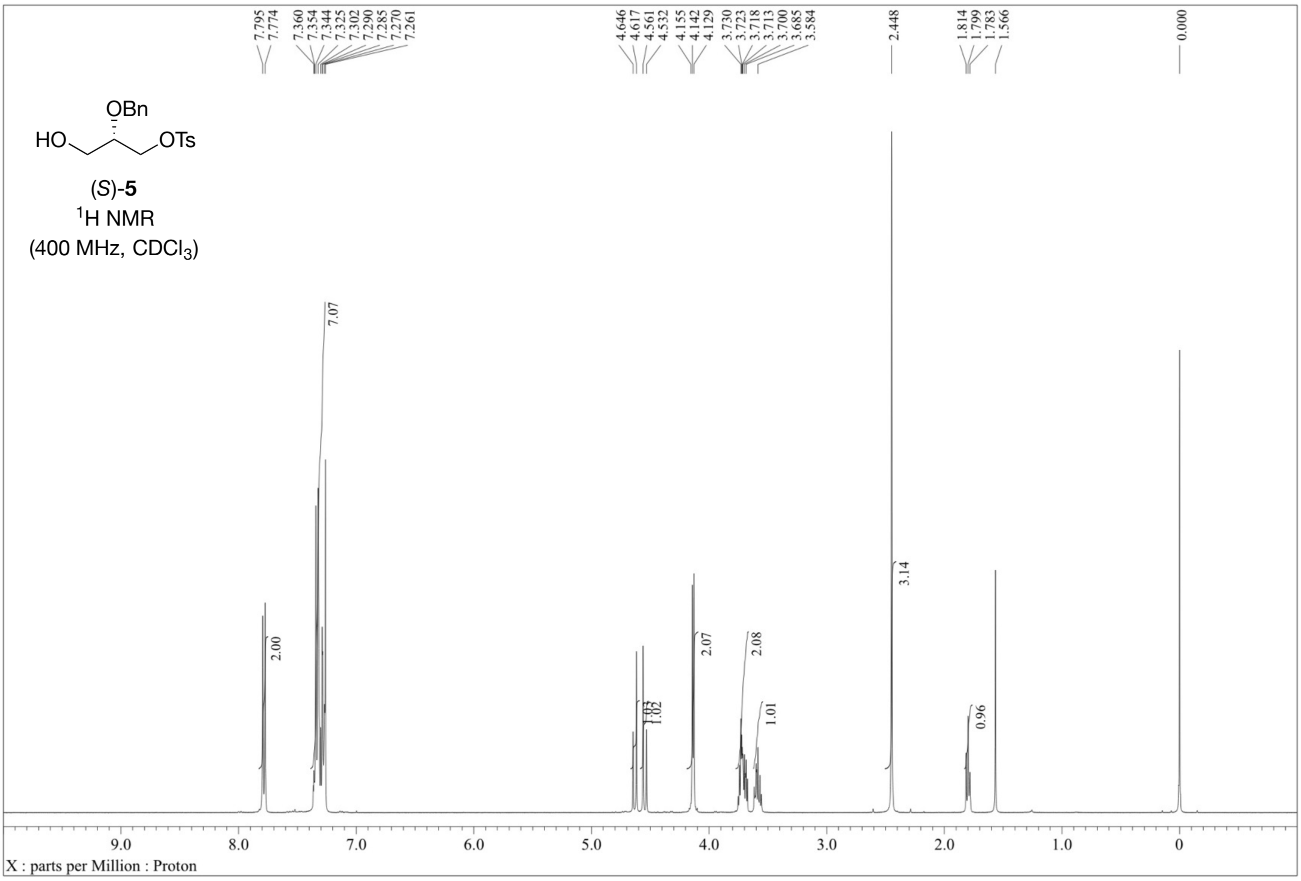

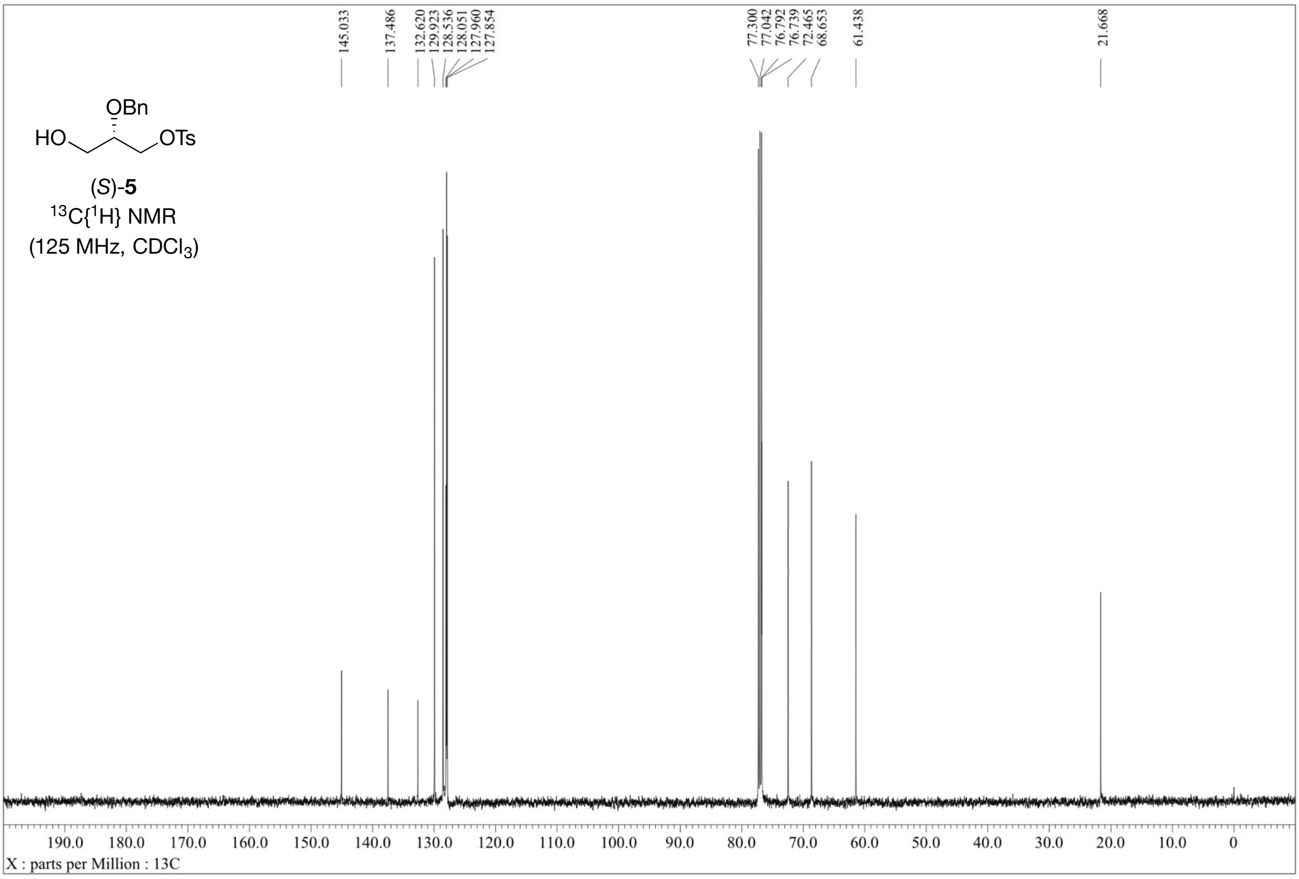

Figure S2. ^{1}H and $^{13}C\{^{1}H\}$ NMR spectra of (*S*)-**5**.

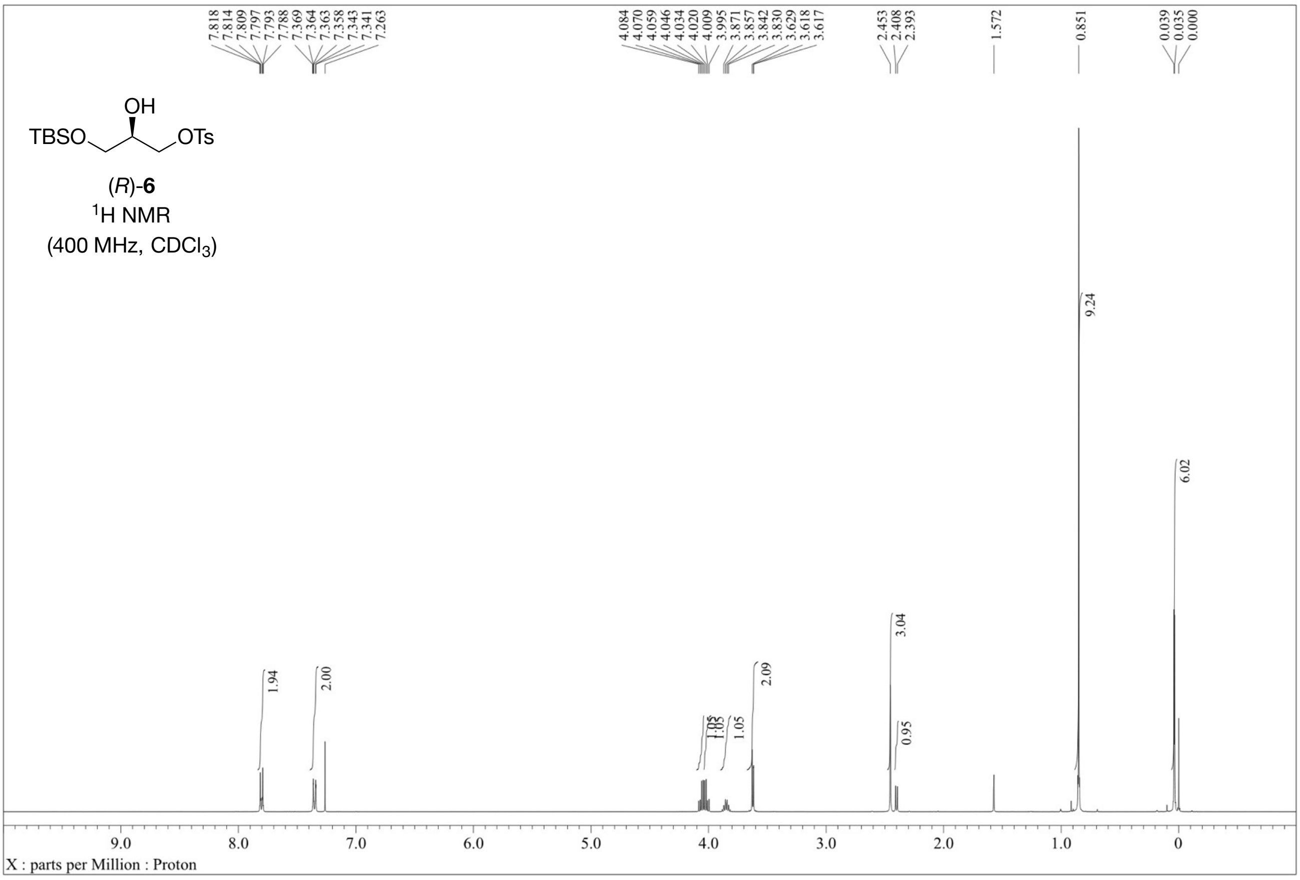

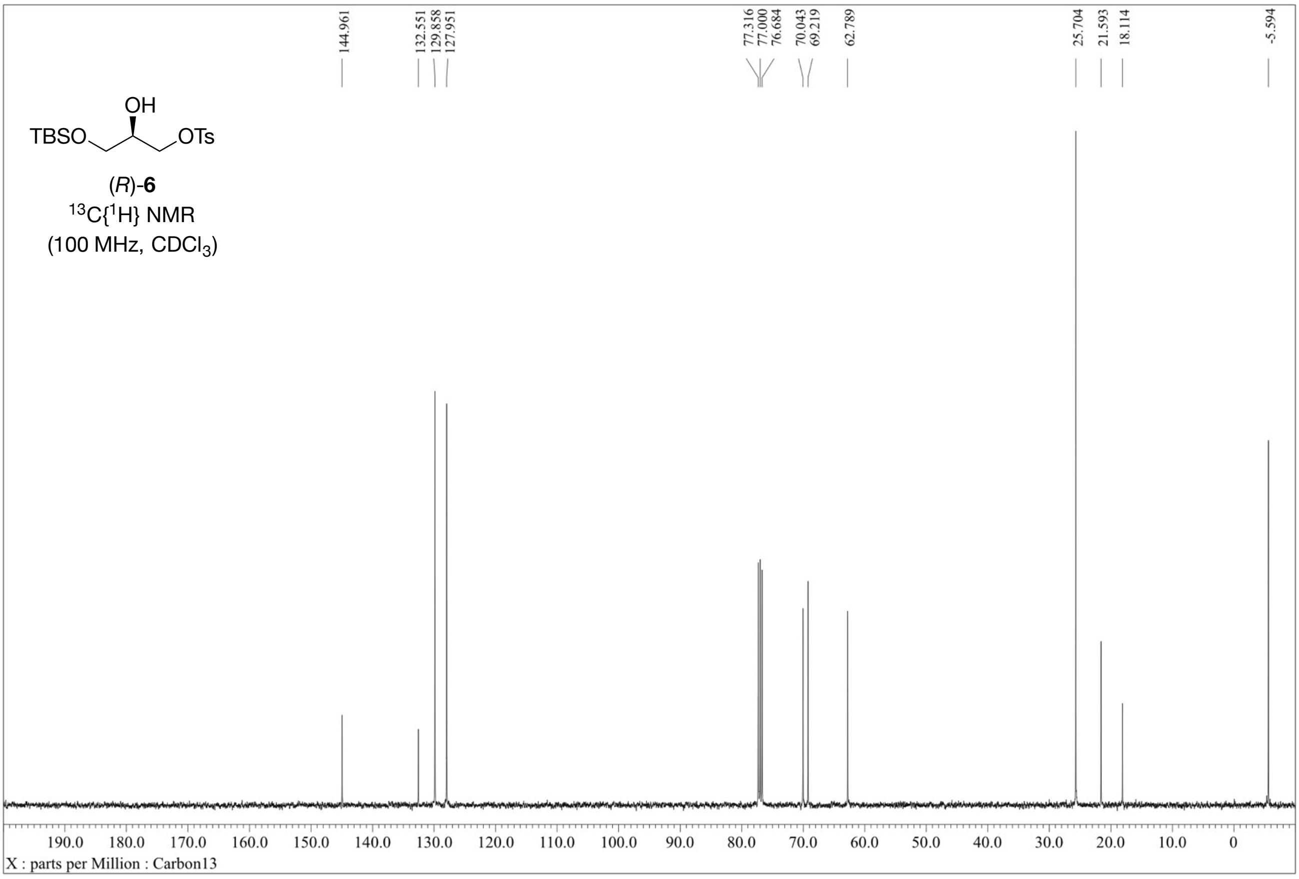

Figure S3. ^{1}H and $^{13}C\{^{1}H\}$ NMR spectra of (*R*)-**6**.

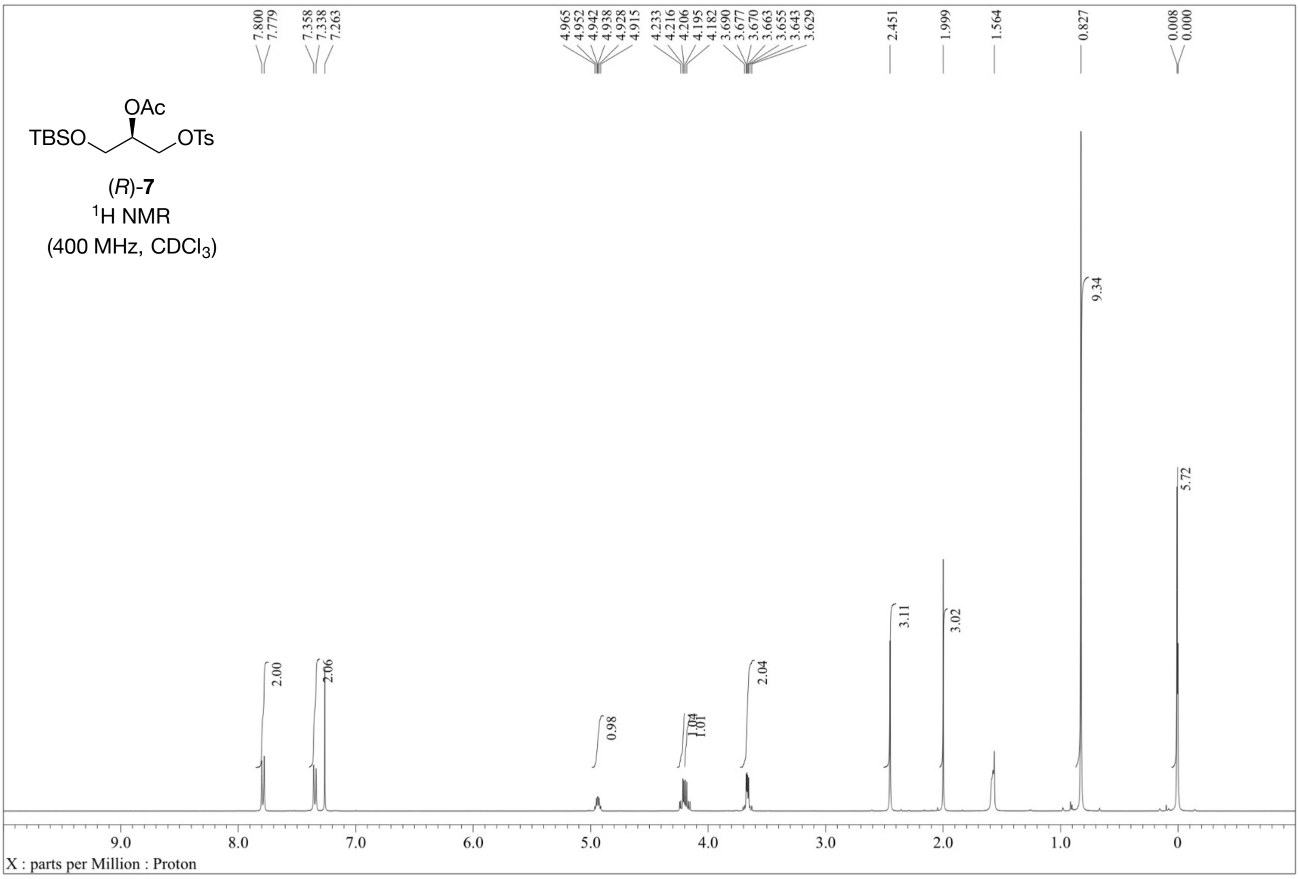

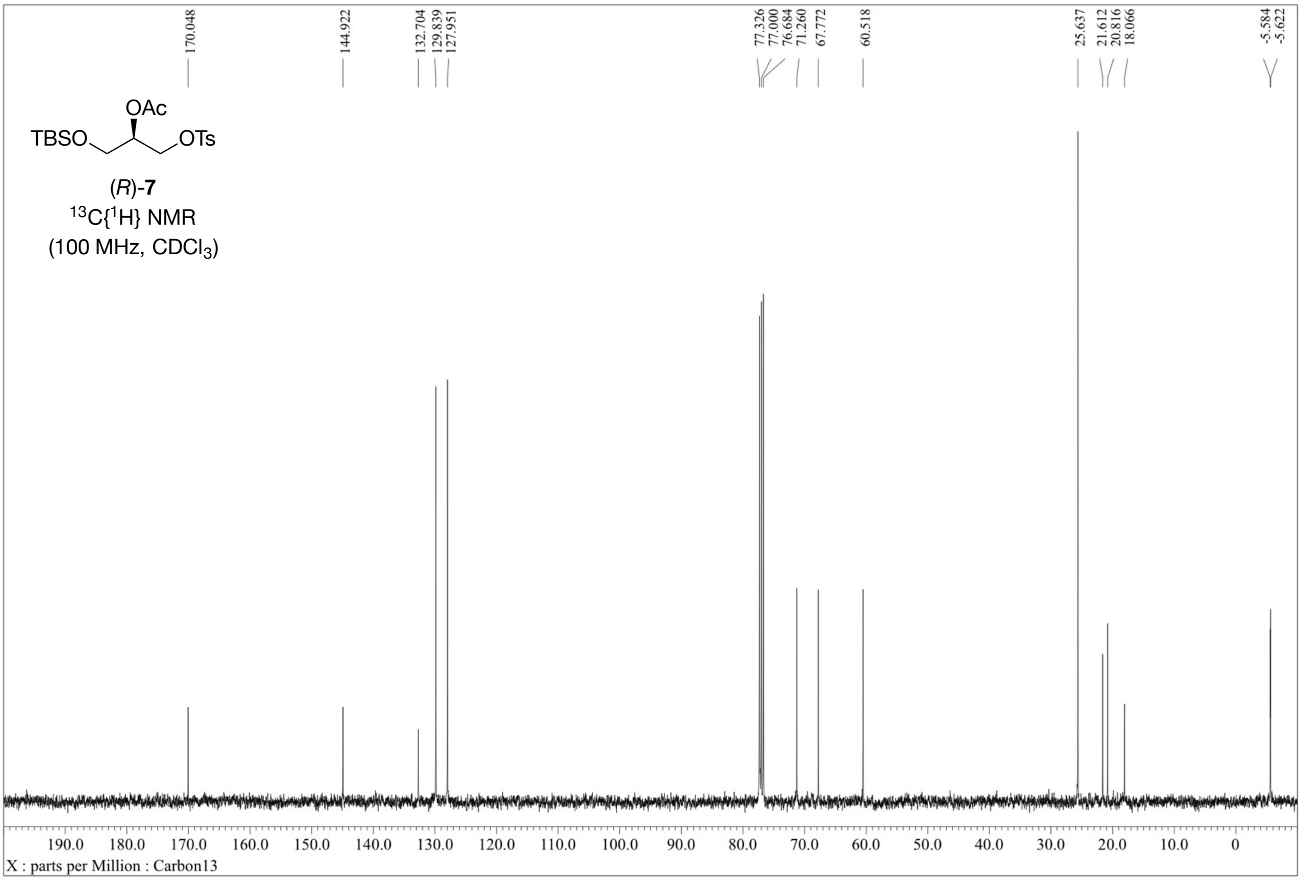

Figure S4. ^{1}H and ^{13}C{^{1}H} NMR spectra of (*R*)-**7**.

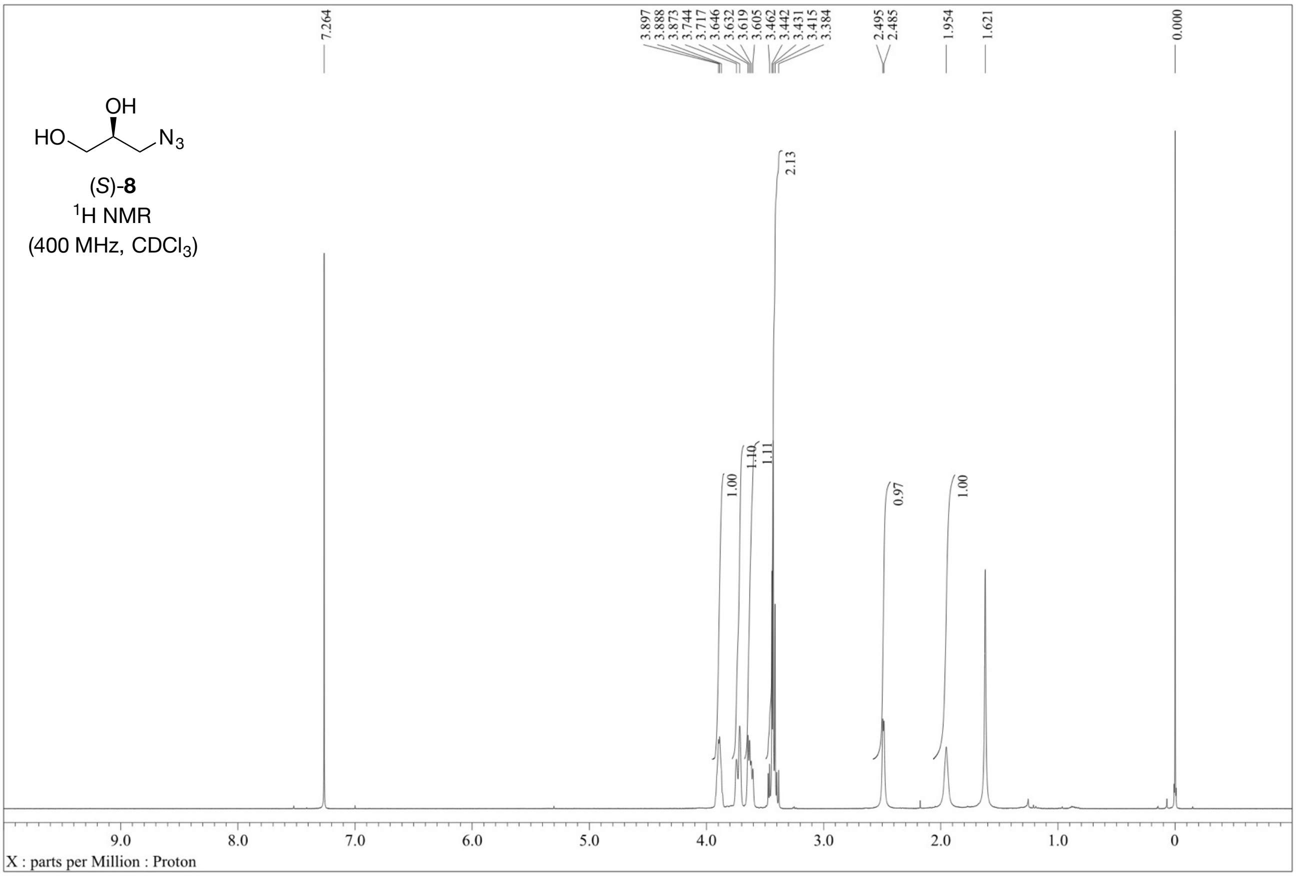

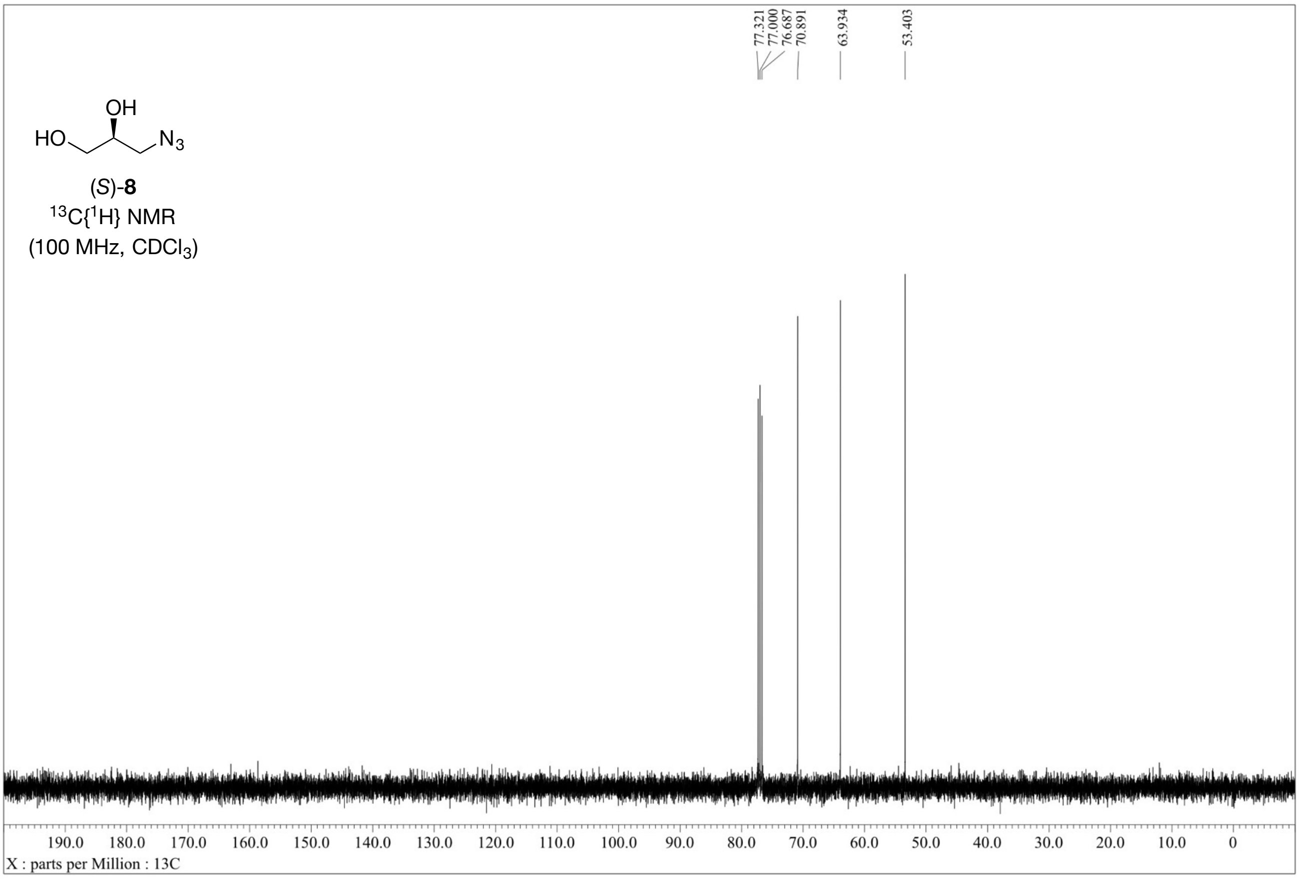

Figure S5. ^{1}H and ^{13}C{^{1}H} NMR spectra of (*S*)-**8**.

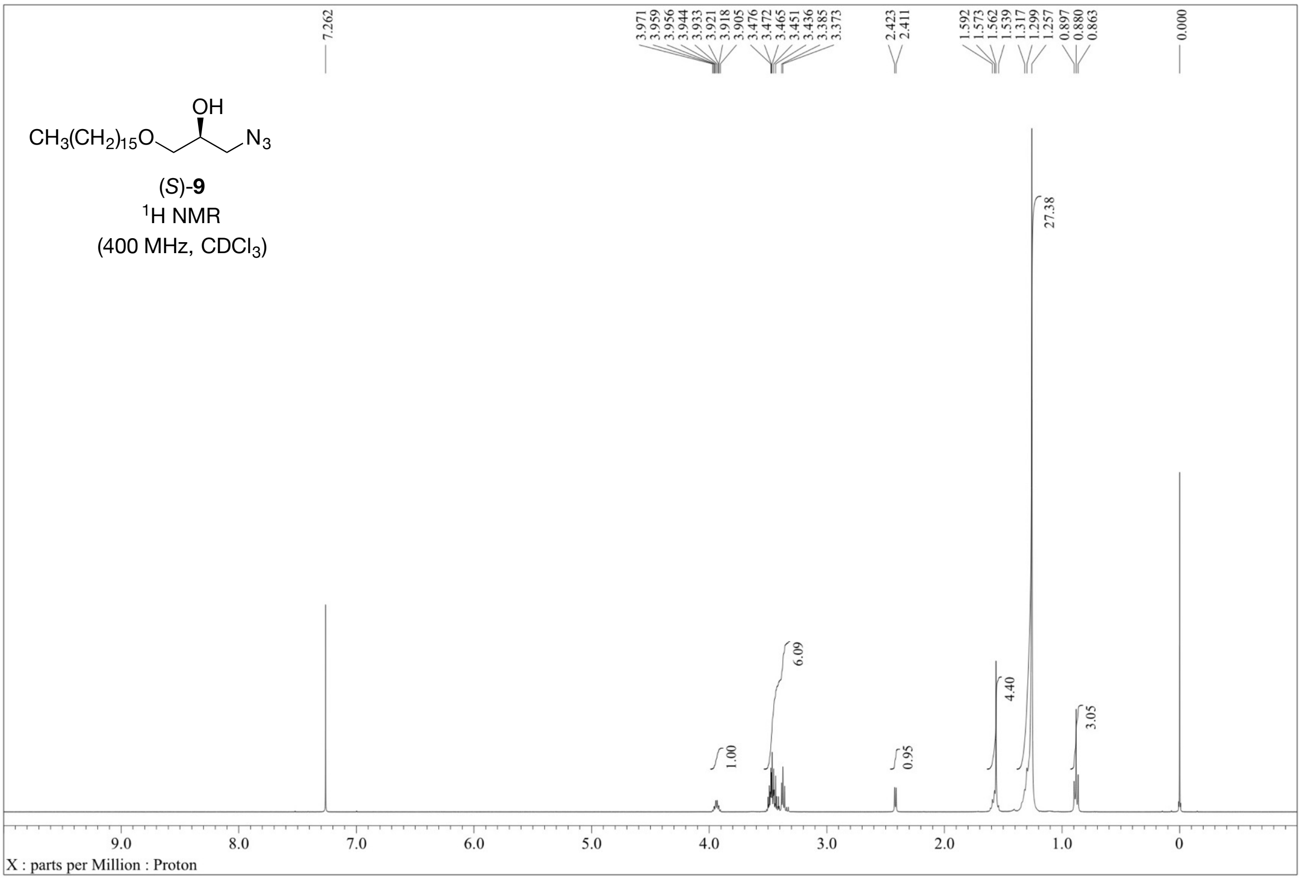

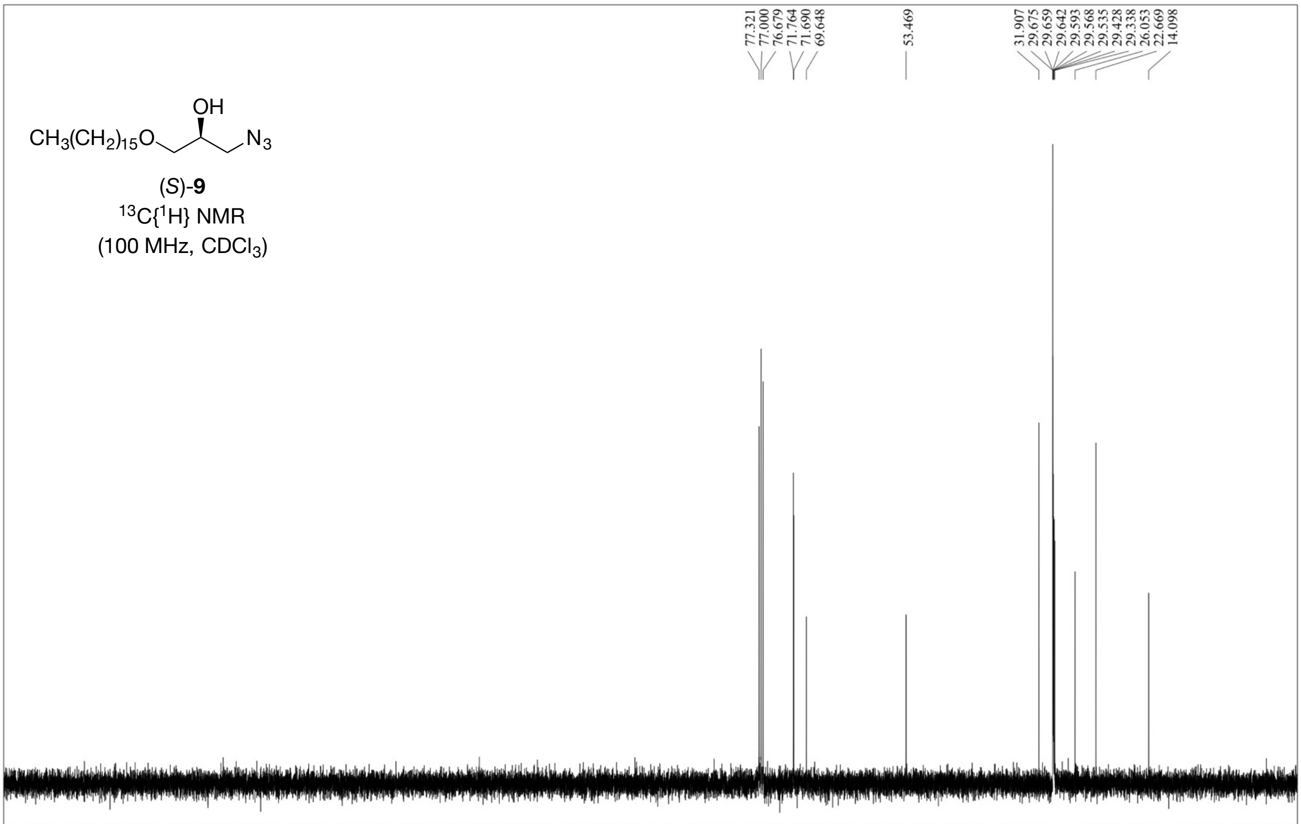

Figure S6. ^{1}H and ^{13}C{^{1}H} NMR spectra of (*S*)-**9**.

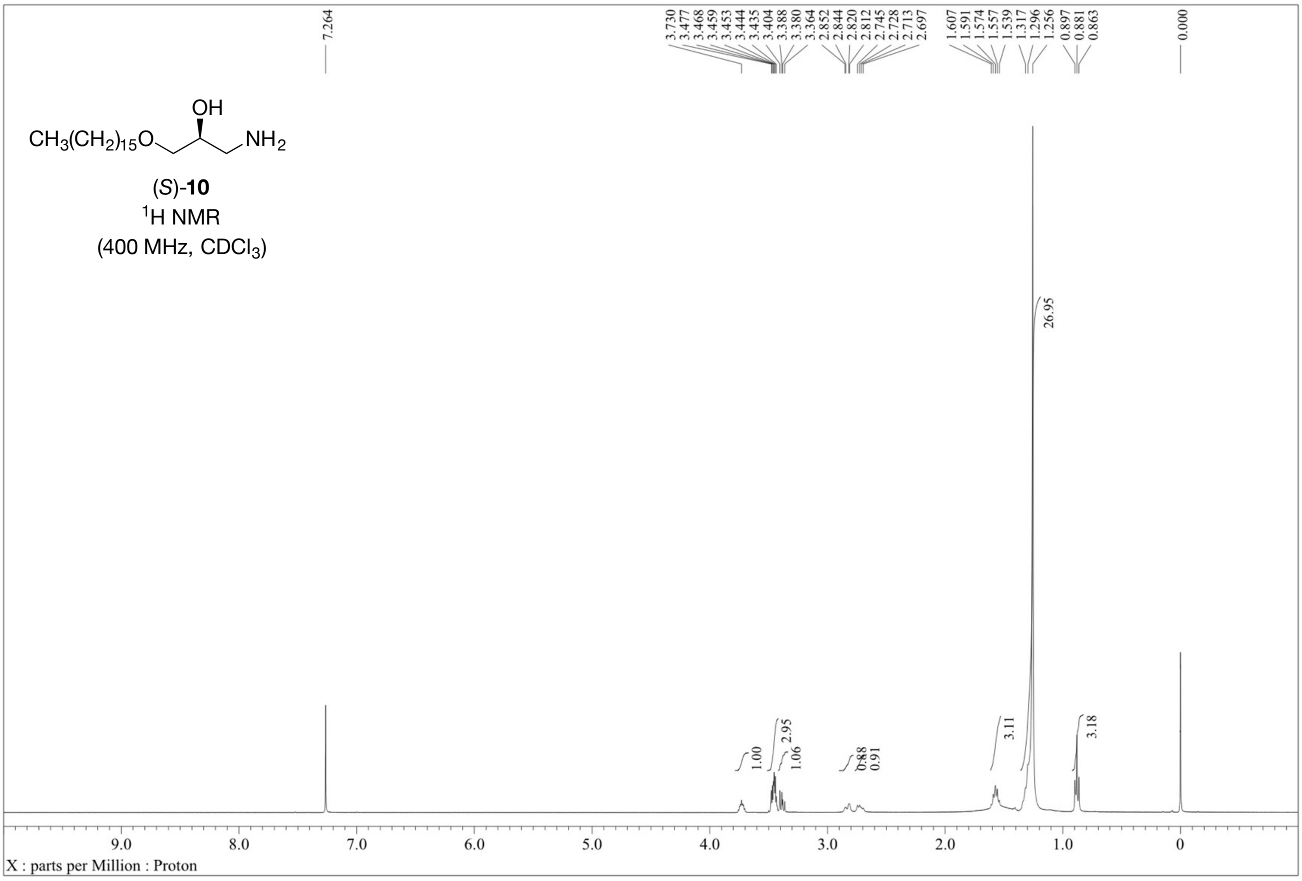

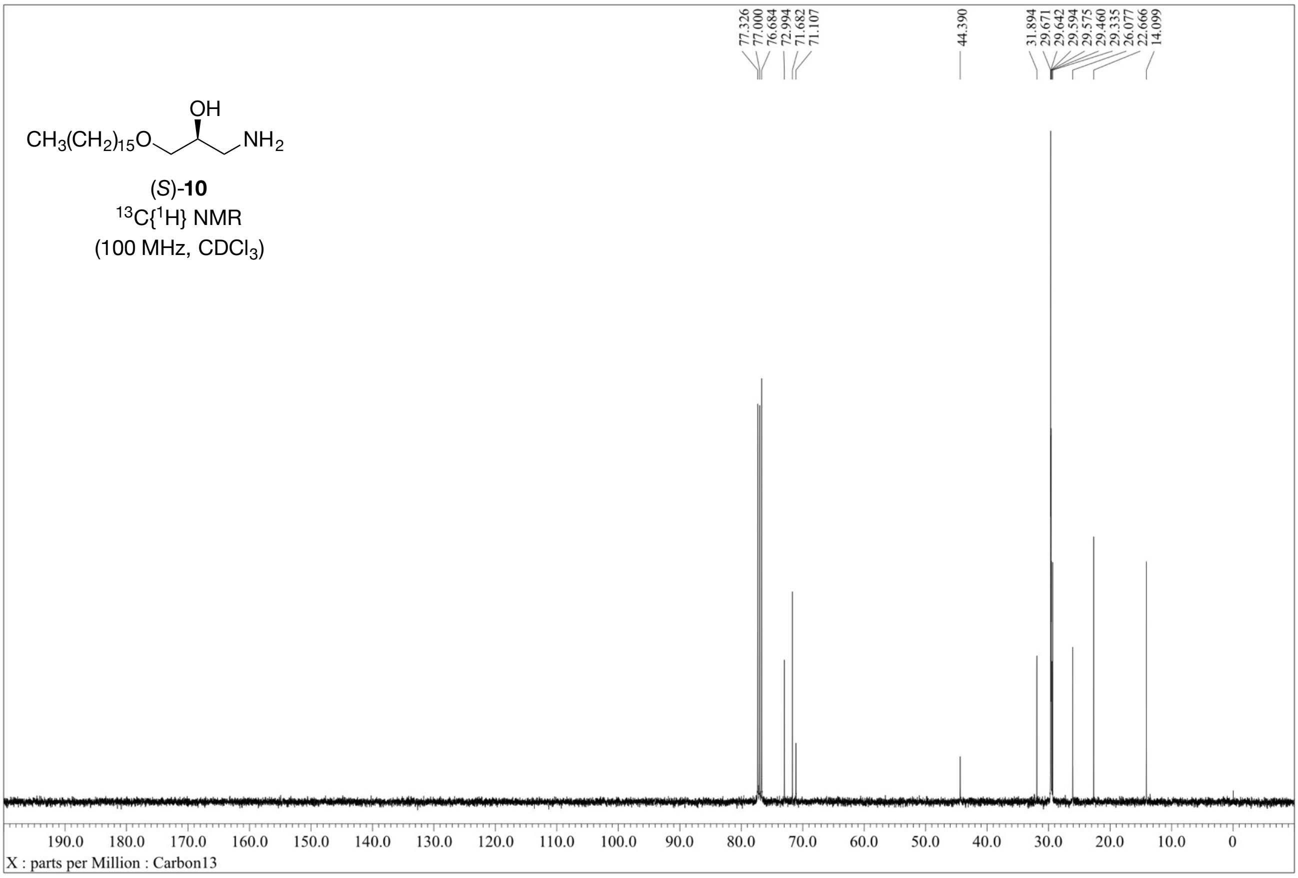

Figure S7. ^{1}H and $^{13}C\{^{1}H\}$ NMR spectra of (*S*)-**10**.

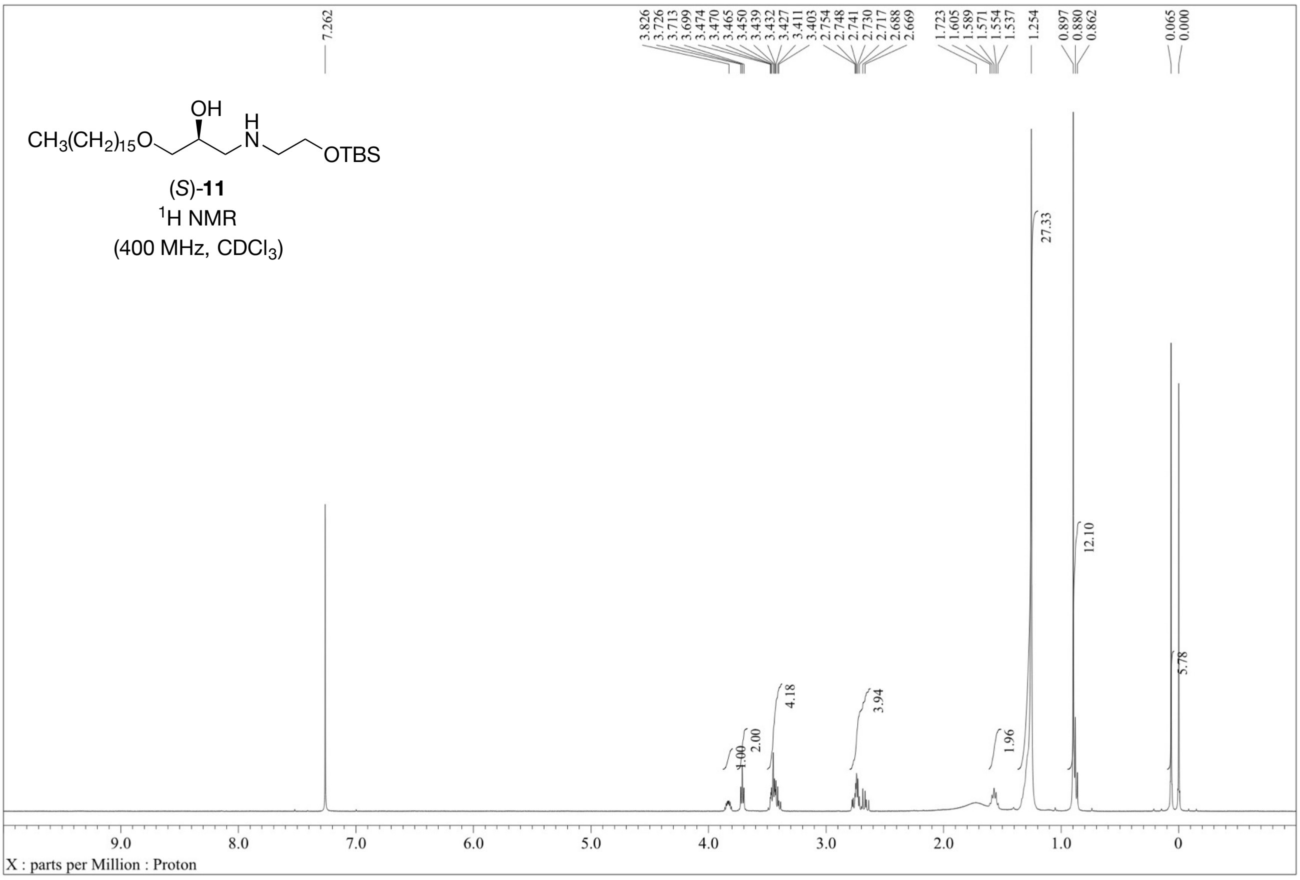

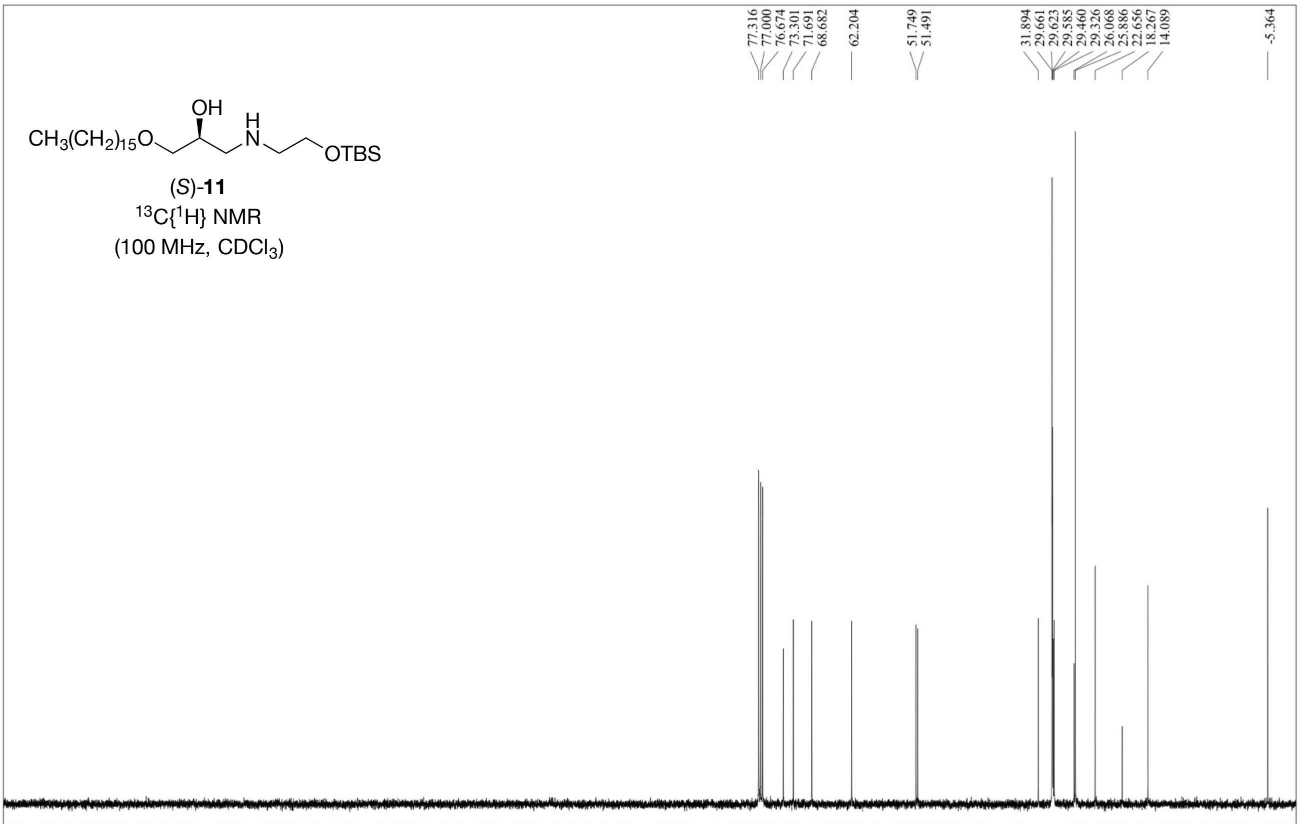

Figure S8. ^{1}H and $^{13}C\{^{1}H\}$ NMR spectra of (*S*)-**11**.

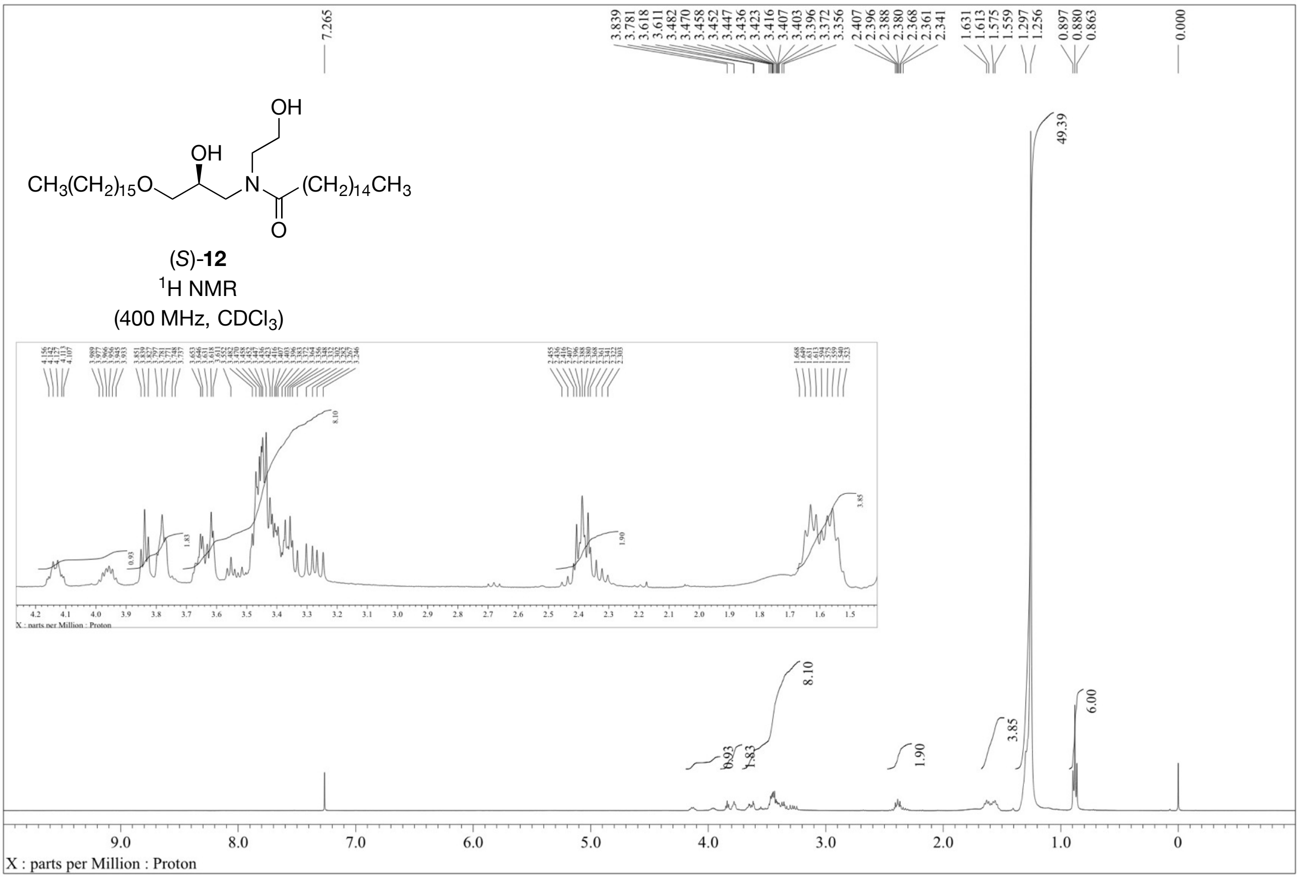

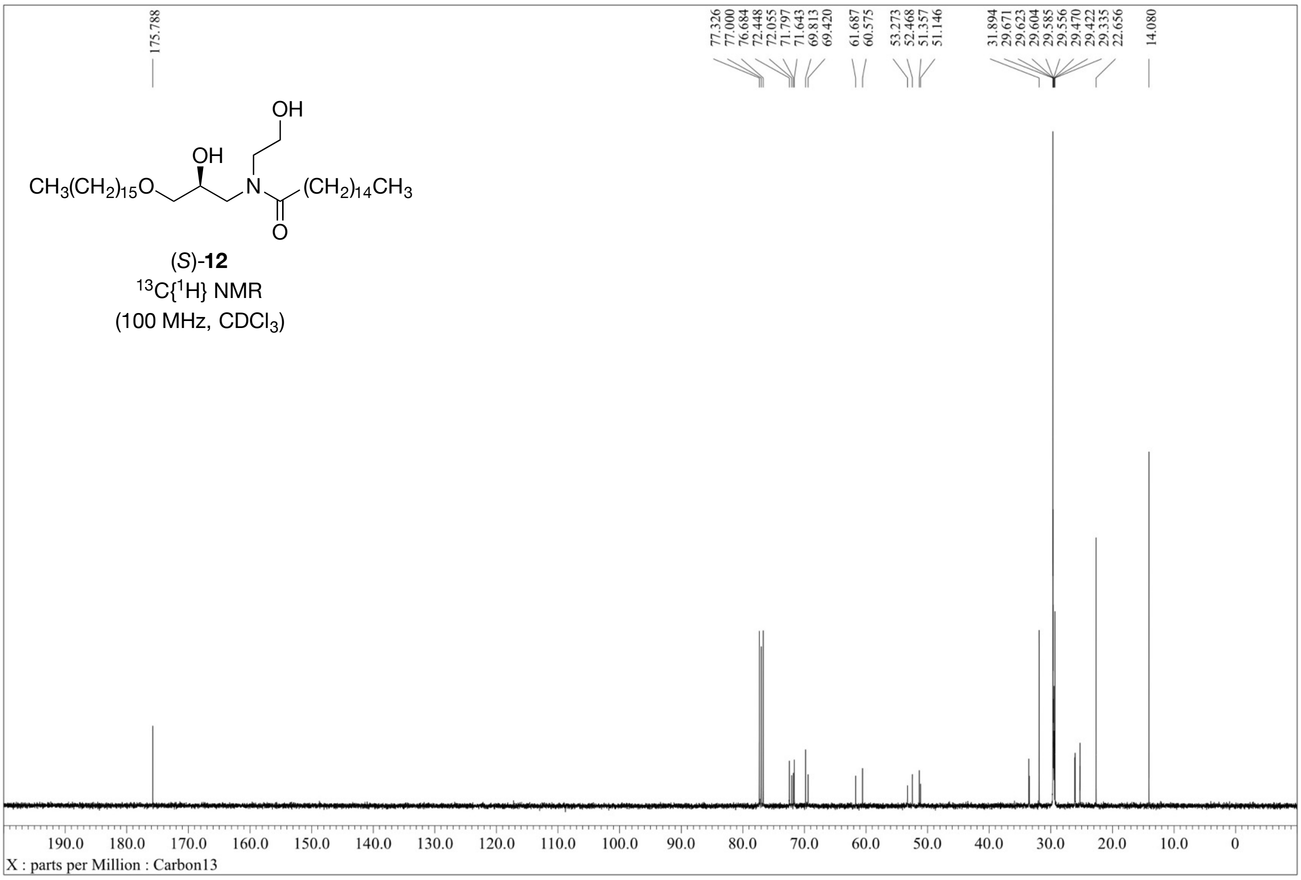

Figure S9. ^{1}H and ^{13}C{^{1}H} NMR spectra of (*S*)-**12**.

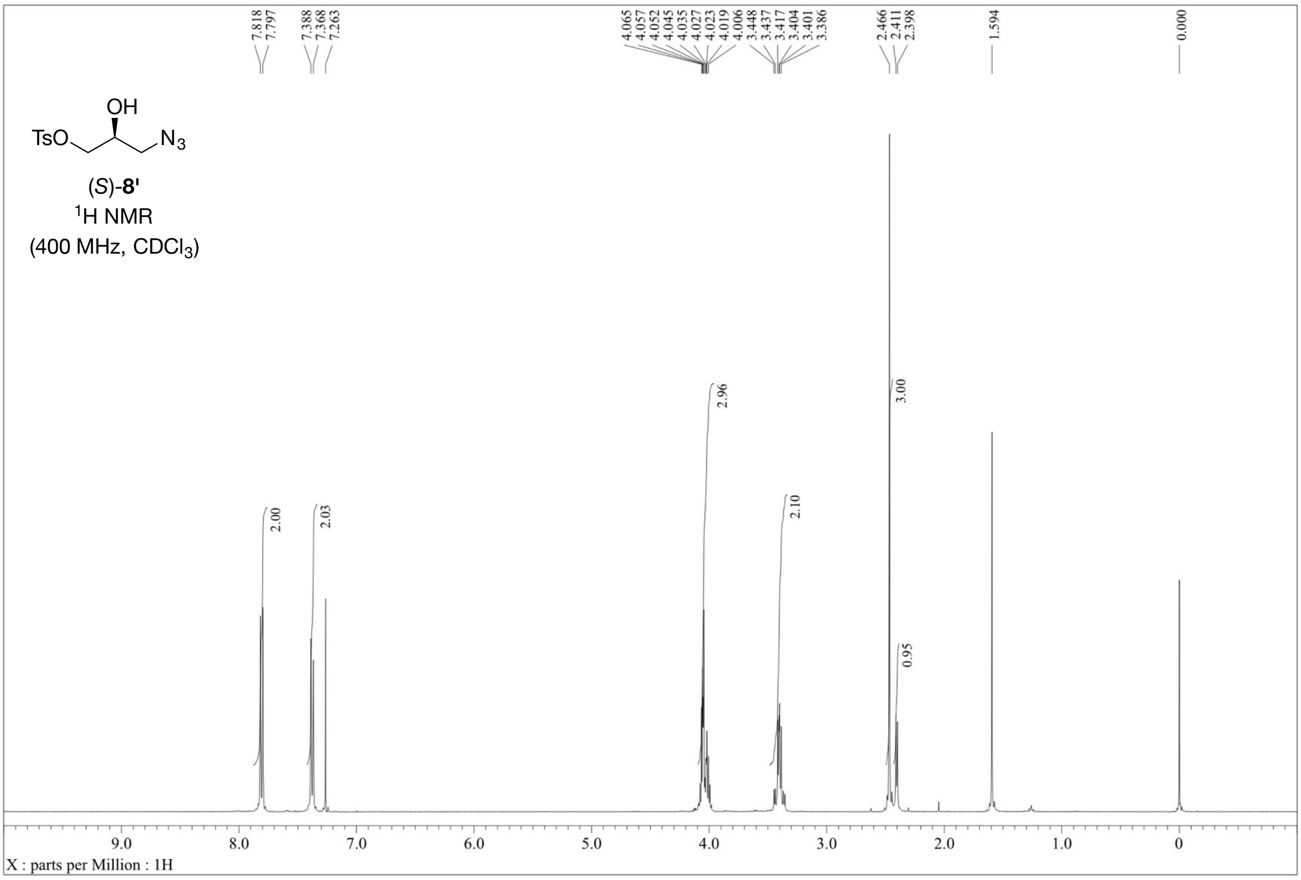

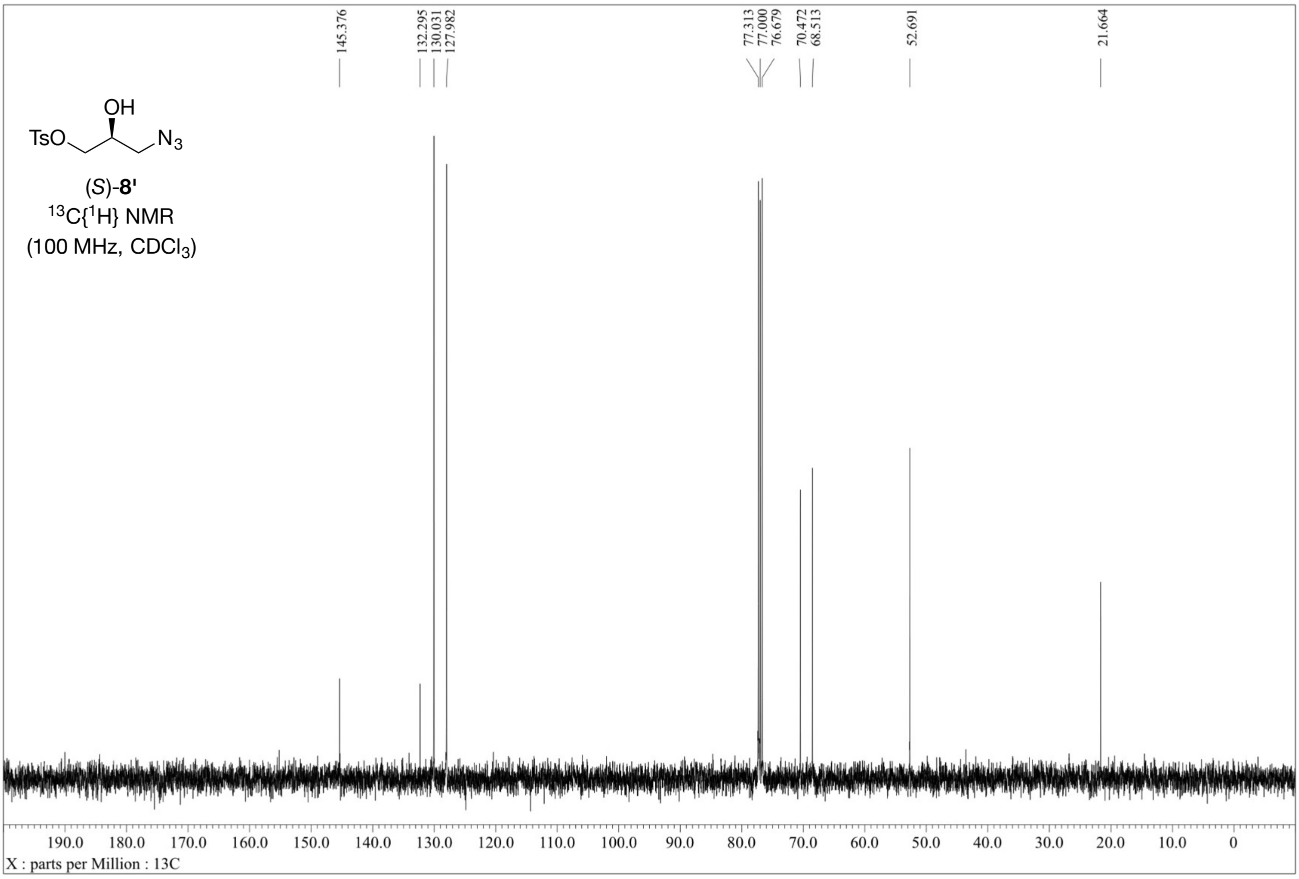

Figure S10. ^{1}H and $^{13}C\{^{1}H\}$ NMR spectra of (*S*)-**8’**.

2. Chiral HPLC chromatograms

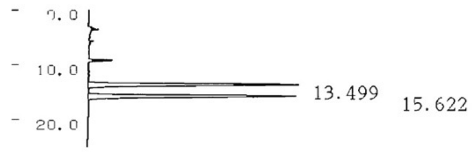

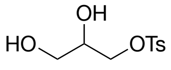

rac-**2**

** CALCULATION REPORT **

CH	PKNO	TIME	AREA	HEIGHT	MK	IDNO	CONC	NAME
1	20	13.499	242404	8692			50.0419	
	21	15.622	241998	8531			49.9581	
		TOTAL	484402	17223			100	

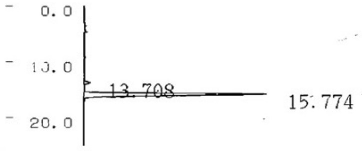

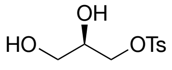

(*R*)-**2**

** CALCULATION REPORT **

CH	PKNO	TIME	AREA	HEIGHT	MK	IDNO	CONC	NAME
1	16	13.708	6691	251			3.08	
	17	15.774	210540	7383			96.9199	
		TOTAL	217231	7634			100	

** GROUPING BY IDNO **

Figure S11. Chiral HPLC traces for *rac*-**2** and (*R*)-**2**.

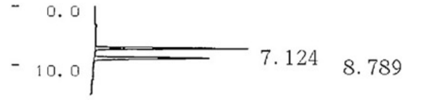

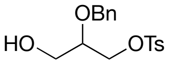

rac-**5**

** CALCULATION REPORT **

CH	PKNO	TIME	AREA	HEIGHT	MK	IDNO	CONC	NAME
1	6	7.124	42087	2928			50.1157	
	7	8.789	41892	2213			49.8843	
		TOTAL	83979	5141			100	

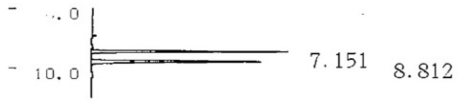

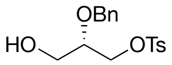

(*S*)-**5**

** CALCULATION REPORT **

CH	PKNO	TIME	AREA	HEIGHT	MK	IDNO	CONC	NAME
1	13	7.151	110950	7496	V		46.6404	
	15	8.812	126934	6459	V		53.3596	
		TOTAL	237884	13955			100	

Figure S12. Chiral HPLC traces for *rac*-**5** and (*S*)-**5**.

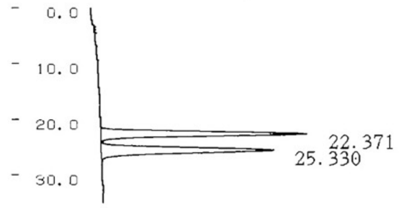

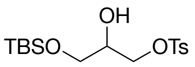

rac-**6**

** CALCULATION REPORT **

CH	PKNO	TIME	AREA	HEIGHT	MK	IDNO	CONC	NAME
1	14	22.371	235557	4191			49.6637	
	15	25.33	238746	3505	V		50.3363	
	TOTAL		474303	7696			100	

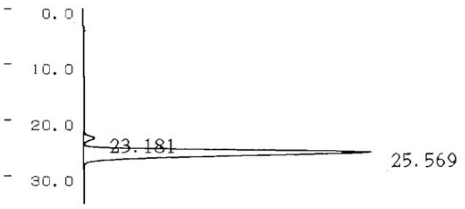

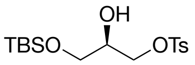

(*R*)-**6**

** CALCULATION REPORT **

CH	PKNO	TIME	AREA	HEIGHT	MK	IDNO	CONC	NAME
1	71	23.181	13014	224	V		2.9149	
	72	25.569	433432	5842	SV		97.0851	
	TOTAL		446445	6066			100	

Figure S13. Chiral HPLC traces for *rac*-**6** and (*R*)-**6**.

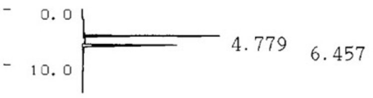

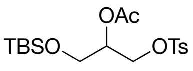

rac-**7**

** CALCULATION REPORT **

CH	PKNO	TIME	AREA	HEIGHT	MK	IDNO	CONC	NAME
1	3	4.779	105843	10376	V		49.1325	
	5	6.457	109581	7478	V		50.8675	
	TOTAL		215423	17855			100	

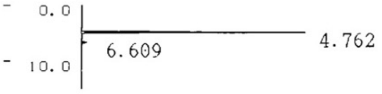

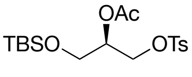

(*R*)-**7**

** CALCULATION REPORT **

CH	PKNO	TIME	AREA	HEIGHT	MK	IDNO	CONC	NAME
1	4	4.762	328293	36447	V		96.8266	
	5	6.609	10760	801			3.1734	
	TOTAL		339053	37248			100	

Figure S14. Chiral HPLC traces for *rac*-**7** and (*R*)-**7**.

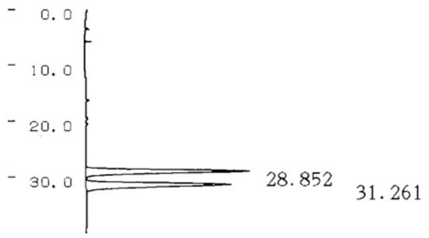

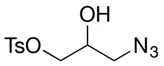

rac-**8'**

** CALCULATION REPORT **

CH	PKNO	TIME	AREA	HEIGHT	MK	IDNO	CONC	NAME
1	16	28.852	287708	6647			49.821	
	17	31.261	289775	5898	V		50.179	
		TOTAL	577483	12545			100	

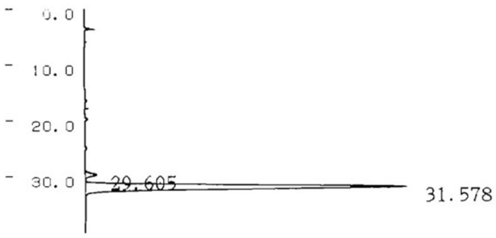

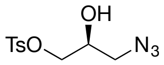

(*S*)-**8'**

** CALCULATION REPORT **

CH	PKNO	TIME	AREA	HEIGHT	MK	IDNO	CONC	NAME
1	56	29.605	9369	227	V		2.7782	
	57	31.578	327869	6510	V		97.2218	
		TOTAL	337238	6737			100	

Figure S15. Chiral HPLC traces for *rac*-**8’** and (*R*)-**8’**.
